# Cyclin‐dependent kinase 12 deficiency reprogrammes cellular metabolism to alleviate ferroptosis potential and promote the progression of castration‐resistant prostate cancer

**DOI:** 10.1002/ctm2.1678

**Published:** 2024-05-12

**Authors:** Haozhe Zhang, Yi Zhou, Yating Feng, Wenli Hou, Yafei Chen, Zengzhen Xing, Yifan Zhang, Qiang Wei, Yu Yin, Ju Guo, Hailiang Hu

**Affiliations:** ^1^ Department of Biochemistry School of Medicine Southern University of Science and Technology Shenzhen China; ^2^ Department of Urology Nanfang Hospital, Southern Medical University Guangzhou China; ^3^ Department of Pathology The First Affiliated Hospital of Anhui Medical University Hefei China; ^4^ Department of Urology The First Affiliated Hospital of Nanchang University Nanchang China; ^5^ Key University Laboratory of Metabolism and Health of Guangdong Southern University of Science and Technology Shenzhen China

**Keywords:** CDK12, CRPC, ferroptosis, metabolism

## Abstract

**Background:**

Cyclin‐dependent kinase 12 (CDK12)‐deficient prostate cancer defines a subtype of castration‐resistant prostate cancer (CRPC) with a poor prognosis. Current therapy, including PARP inhibitors, shows minimal treatment efficacy for this subtype of CRPC, and the underlying mechanism remains elusive.

**Methods:**

Based on bioinformatics analysis, we evaluated the relationship between CDK12 deficiency and prostate cancer patient's prognosis and treatment resistance. Furthermore, we used CRISPR‐Cas9 technology and mass spectrometry‐based metabolomic profiling to reveal the metabolic characteristics of CDK12‐deficient CRPC. To elucidate the specific mechanisms of CDK12 deficiency‐mediated CRPC metabolic reprogramming, we utilized cell RNA‐seq profiling and other molecular biology techniques, including cellular reactive oxygen species probes, mitochondrial function assays, ChIP‐qPCR and RNA stability analyses, to clarify the role of CDK12 in regulating mitochondrial function and its contribution to ferroptosis. Finally, through in vitro drug sensitivity testing and in vivo experiments in mice, we identified the therapeutic effects of the electron transport chain (ETC) inhibitor IACS‐010759 on CDK12‐deficient CRPC.

**Results:**

CDK12‐deficient prostate cancers reprogramme cellular energy metabolism to support their aggressive progression. In particular, CDK12 deficiency enhanced the mitochondrial respiratory chain for electronic transfer and ATP synthesis to create a ferroptosis potential in CRPC cells. However, CDK12 deficiency downregulated ACSL4 expression, which counteracts the lipid oxidation stress, leading to the escape of CRPC cells from ferroptosis. Furthermore, targeting the ETC substantially inhibited the proliferation of CDK12‐deficient CRPC cells in vitro and in vivo, suggesting a potential new target for the therapy of CDK12‐deficient prostate cancer.

**Conclusions:**

Our findings show that energy and lipid metabolism in CDK12‐deficient CRPC work together to drive CRPC progression and provide a metabolic insight into the worse prognosis of CDK12‐deficient prostate cancer patients.

**Key points:**

- CDK12 deficiency promotes castration‐resistant prostate cancer (CRPC) progression by reprogramming cellular metabolism.
- CDK12 deficiency in CRPC leads to a more active mitochondrial electron transport chain (ETC), ensuring efficient cell energy supply.
- CDK12 phosphorylates RNA Pol II to ensure the transcription of ACSL4 to regulate ferroptosis.
- Mitochondrial ETC inhibitors exhibit better selectivity for CDK12‐deficient CRPC cells, offering a promising new therapeutic approach for this subtype of CRPC patients.

## Graphical abstract

- CDK12 deficiency reprograms prostate tumor cell metabolism, promotes tumor progression while demonstrating therapeutic targets.
- Identified the regulation of CDK12 on ACSL4 expression as a novel mechanism for controlling ferroptosis.

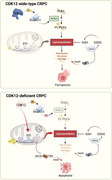

## 1 BACKGROUND

Cancer cells can reprogramme the cellular metabolism to adapt and survive under different conditions and maintain the growth and spread of tumours.^1^, ^2^ One of the key characteristics of cancer is the dysregulation of energy metabolism^3^; a well‐known example is the Warburg effect, where cancer cells show abnormal glucose metabolism.^4^ Mitochondria, known as the energy factory of cells, play a vital role in energy metabolism, and the mitochondrial electron transport chain (ETC) consists of five large complexes that maintain mitochondrial homeostasis and provide adenosine 5′‐triphosphate (ATP) for cell proliferation, migration and other behaviours.^5^, ^6^ Cancer cells have an altered energy metabolism that can produce reactive oxygen species (ROS) during the chemical reactions and electron transfers in the ETC, causing oxidative stress.^7^, ^8^, ^9^ Excessive oxidative stress can disrupt cellular homeostasis and trigger programmed cell death processes, including apoptosis and ferroptosis.^9^, ^10^, ^11^ However, the mechanisms by which cancer cells manage oxidative stress during ATP production still need to be fully understood. Disrupting this delicate balance may potentially lead to the destruction of cancer cells and open up new avenues for cancer treatment.

Cyclin‐dependent kinase 12 (CDK12) is a member of the CDK family and plays a critical role in regulating the transcription process,^12^, ^13^ which interacts with cyclin K to activate its kinase activity.^14^ It influences transcription elongation and the utilization of alternative polyadenylation sites through serine phosphorylating the carboxy‐terminal domain (CTD) of RNA polymerase II (RNA Pol II).^15^, ^16^ Biallelic mutation of CDK12 leads to defects in the Drosophila nervous system and causes murine embryonic death, highlighting the critical role of CDK12 in development and physiology.^17^, ^18^ CDK12 mutations are found in many types of cancer,^19^, ^20^, ^21^, ^22^, ^23^ but the exact function of CDK12 in the initiation and progression of cancer is not fully understood. CDK12 has been classified as a DNA damage repair (DDR)‐related gene because its inactivation affects DNA double‐strand break homologous recombination (HR) repair‐related genes’ expression.^15^, ^16^ PARP inhibitors (PARPi) targeting HR repair defects have been used in clinical trials of patients with CDK12‐inactivated prostate cancer.^24^, ^25^ However, PARPi has not achieved satisfactory efficacy for CDK12‐mutated patients with prostate cancer.^26^, ^27^ In addition, this group of patients has a poorer prognosis with other conventional therapies, such as hormone therapy and chemotherapy.^27^, ^28^, ^29^ While CDK12 inactivation results in focal tandem duplications in the genome of prostate cancer cells and generates a large number of neoantigens, which could make patients of this subtype ideal candidates for immunotherapy, further clinical testing is needed to determine the efficacy of this treatment.^19^ Thus, CDK12‐deficient prostate cancer patients continue to pose a most significant challenge for effective treatment in clinical practice.

In this study, we revealed that the loss‐of‐function mutations of CDK12 lead to metabolic reprogramming in castration‐resistant prostate cancer (CRPC) cells, increasing the mitochondrial ETC activity to ensure the cellular energy supply. However, this process simultaneously raises oxidative stress, increasing the risk of ferroptosis.^9^, ^30^ To offset this risk, CDK12 deficiency downregulates the expression of acyl‐CoA synthetase long‐chain family member 4 (ACSL4), which reduces the incorporation of polyunsaturated fatty acid (PUFA) into the cell membrane and thus helps cancer cells avoid ferroptosis. Furthermore, due to the greater dependence of CDK12‐deficient CRPC cells on the ETC activity, pharmacologically targeting the mitochondrial ETC disrupts the energy supply and causes mitochondrial depolarization that makes CDK12‐deficient CRPC cells more susceptible to ETC inhibition‐induced apoptosis.

## 2 METHODS

### 2.1 Cell culture

C4‐2 and PC3 human prostate cancer cell lines, A549 human lung cancer cell line, A375 human melanoma cell line, HCT116 human colorectal cancer cell line and HEK 293T human embryonic kidney cell line were obtained from the American Type Culture Collection. C4‐2, PC3, A549 and HCT116 cells were cultured in Roswell Park Memorial Institute (RPMI)−1640 medium (Gibco, cat. #11875119), while A375 cells and HEK 293T cells were cultured in Dulbecco's Modified Eagle Medium (DMEM, Gibco, cat. #10569044). Both media were supplemented with 10% foetal bovine serum (FBS, ExCell, cat. #FCS500) and 100 units/mL penicillin/streptomycin (Gibco, cat. #15140122). The cells were incubated in a 5% (vol/vol) CO_2_ and 95% (vol/vol) air incubator (ThermoFisher Co.) at 37°C.

### 2.2 CRISPR/Cas9‐mediated CDK12 knockout

To establish a CDK12 knockout stable cell line, we used the CRISPR/Cas9 technology. Single‐guided RNA (sgRNA) sequences (5′‐CGGCGACGTCAGAGACAAAG‐3′) were generated by the Benchling (benchling.com). Annealed double‐stranded sgRNA oligos were ligated to the lentiCRISPR v2 (deposited by Feng Zhang Lab to Addgene cat. #52961) at a ribonucleoprotein complex expressing Cas9 and sgRNA. The transfer plasmid was transfected into HEK 293T cells with packaging plasmid psPAX.2 (deposited by Trono Lab to Addgene cat. #12260) and envelope plasmid pMD2.G (deposited by Trono Lab to Addgene cat. #12259) to produce an infectious transgenic lentivirus. After the change in medium and a brief incubation period, a supernatant containing the virus was collected and centrifuged to concentrate the virus. After using the virus to infect, cells were selected by puromycin (Selleck, cat. #S7417) for more than 2 weeks. The population that showed no target protein expression was confirmed by western blot.

### 2.3 Metabolite extraction and mass spectrometry

1 × 10^8^ cells were placed in 15 cm plates. After 24 h, the medium was replaced with 20 mL of fresh medium per plate, and the cells were incubated for another 24 h. The medium was then aspirated, and 80% (vol/vol) high‐performance liquid chromatography grade methanol/water was added to each plate. The plates were immediately placed on dry ice and then transferred to a −80°C freezer for 15 min to inactivate enzymes. The cells were then scraped into the extraction solvent, and after centrifugation for 10 min, the supernatant was transferred into a new Eppendorf tube. The samples were then dried using a speed vacuum at room temperature; the dried metabolite pellets were stored at −80°C. Before mass spectrum analysis, the dried metabolite pellets were dissolved in mobile phase A (.03% formic acid in water, 1 μL mobile phase A per 1 × 10^6^ cells). Liquid chromatography‐mass spectrometry (LC‐MS) and data analysis were conducted following the protocol described previously.^31^ Specifically, the mass‐to‐charge ratios (m/z), retention times and signal intensity values of the ions of metabolite ions, as well as secondary fragment ions obtained from the parsing of the raw data, were matched with the spectral information of primary and secondary metabolites in the Human Metabolome Database to determine the set of detected metabolites, with three biological replicates for each sample. The data were normalized to the number of cells. The metabolite with an average concentration lower than 1000 was discarded.

### 2.4 RNA‐seq analysis

RNA was extracted from cell lines using RNeasy mini kits (Qiagen, cat. #74104). RNA integrity was assessed and determined using Nanodrop (ThermoFisher Co.). A total of 3 μg of RNA per sample was used for analysis. Sequencing sampling was performed from paired‐end replications. RNA‐seq was performed on an Illumina HiSeq 3000 platform (Illumina Co.). Reads were aligned to the human genome GRCh38. Gene differential expression analysis was done using the differentially expressed genes (DEG)‐Seq2 R package.

### 2.5 Luminescent cell viability assay

According to the manufacturer's instructions, 3000 cells were seeded in 96‐well plates for each well, after 24 h, treated with different drugs and dimethyl sulfoxide (DMSO, Solarbio, cat. #D8371) for 48 h, balanced the plates at room temperature for 30 min, 100 μL CellTiter‐Glo (Promega, cat. #G7572) was added to each well, shaken for 5 min. Chemiluminescence was detected using a Microplate Reader (BioTek Co.). All data were normalized using DMSO groups, data were pre‐processed and line‐fitted, and IC_50_ was calculated using GraphPad Prism 10 (GraphPad‐prism. cn).

### 2.6 Glutathione and glutathione oxidized level test

A Glutathione Assay Kit (Beyotime, cat. #S0053) assessed the relative glutathione (GSH) and glutathione oxidized (GSSG) concentration. Briefly, after the reduction of GSSG to GSH by glutathione reductase, GSH reacts with the chromogenic substrate DTNB (5,5′‐Dithiobis (2‐nitrobenzoic acid)) to produce yellow TNB (5‐thio‐2‐nitrobenzoic acid) and GSSG. The absorbance characterizes the total glutathione (GSSG + GSH) amount, and the amount of GSSG can be determined after pre‐cleaning of GSH from the sample with a GSH scavenging. According to the manufacturer's instructions, 5 × 10^4^ cells were seeded in 6‐well plates for each well; after 24 h, the cells were washed once with cold PBS (Gibco, cat. #10010023), collect the cells by centrifugation and aspirate the supernatant. Add three times the volume of protein removal reagent and vortex thoroughly. The samples were then subjected to two rapid freeze‐thaws using liquid nitrogen and a water bath at 37°C. The samples were left at 4°C for 5 min and centrifuged at 4°C for 10 min at 10 000 × *g*. The supernatant was taken for total glutathione determination. For GSSG measurement, GSH scavenging was added to the above‐prepared sample, immediately vortex‐mixed, and the reaction was carried out at 25°C for 60 min. Then, add 150 μL glutathione reductase, DTNP mix and 50 μL NADPH solution per well. After reaction for 25 min at room temperature, the yellow product was measured spectrophotometrically at 412 nm using a Microplate Reader (BioTek Co.). Data were processed using GraphPad Prism 10.

### 2.7 Quantitative real‐time PCR

RNA was extracted using RNeasy mini kits (Qiagen, cat. #74104). cDNA synthesis was performed by PrimeScript RT Master Mix (Takara, cat. #RR036A). Quantitative real‐time PCR (qRT‐PCR) was applied on ABI 7500 (Applied Biosystems Co.) using AceQ qPCR SYBR Green Master Mix (Vazyme, cat. #Q111‐02). *ACTB* expression assay was used as an internal control. All qPCRs were determined in triplicate or more. All the primer sequences are shown in Table S1.

### 2.8 Immunoblotting

Total protein was collected by lysing adherent cells with RIPA buffer (Sigma, cat. #R0278) supplemented with phosphatase and protease inhibitors (Thermo Scientific, cat. #78440). Protein concentration was measured using the Bradford reagent (Thermo Scientific, cat. #23236). An equal amount of protein was loaded for immunoblotting. After electrophoresis, the protein was transferred to the PVDF transfer membrane (Merck, cat. #0000240973), followed by a 5% BSA blocking buffer, and incubated overnight with primary antibodies. After washing with TBST (TBS with .1% Tween), anti‐rabbit second antibodies (Cell Signaling Technology, cat. #7074) conjugated with HRP‐conjugated were used to probe the membranes. Samples were developed by Chemiluminescent Substrate (Thermo Scientific, cat. #34577) and exposed by Tanon 5200 Multi‐system (Tanon Co.). Densitometry was performed using ImageJ software (National Institutes of Health), and samples were normalized to internal controls. Primary antibodies used in this study are CDK12 antibody (Cell Signaling Technology, cat. #11973), Phospho‐Rpb1 CTD (Ser2/Ser5) Rabbit mAb (Cell Signaling Technology, cat. #13546), β‐Actin Rabbit mAb (Cell Signaling Technology, cat. #8457), GAPDH Rabbit mAb (Cell Signaling Technology, cat. #2118), α‐Tubulin Rabbit mAb (Cell Signaling Technology, cat. #2125) and recombinant Anti‐FACL4 antibody (Abcam, cat. #EPR8640).

### 2.9 Lipid ROS assay

According to the manufacturer's instructions, 1 × 10^5^ cells were seeded in 6‐well plates for each well, after 24 h, treated with 2 μM RSL3 or DMSO for 12 h, cells were collected and washed with PBS, then incubated in 5 μM BODIPY 581/591 C11 dye reagent (Invitrogen, cat. #D3861) in PBS buffer containing 5% FBS at 37°C, 5% CO_2_ for 30 min. Lipid‐ROS levels were analysed by flow cytometry (Agilent Co.).

### 2.10 Chromatin immunoprecipitation‐qPCR assay

Chromatin immunoprecipitation (ChIP) was performed by following the protocol of the Sonication chip Kit (ABclonal, cat. #RK20258), according to the manufacturer's instructions. In brief, 1 × 10^7^ mCRPC cells treated with 1 μM THZ531 (Selleck, cat. #S6595) or DMSO for 12 h were fixed with 1% paraformaldehyde (PFA, biosharp, cat. #BL908A) and proceeded to washing steps with cold PBS. Then, the cells were harvested and subjected to cellular and nuclear lysis. The whole atomic lysate was sonicated with optimal conditions (1 s pulse on, 1 s pulse off, 15 min, 40 W) to yield 200–700 bp DNA. Twenty‐five microlitres of sheared lysate was aliquoted as input. Four hundred and seventy‐five microlitres of the sheared lysate was subjected to immunoprecipitation by 3 h incubation at 4°C of Rpb1 NTD Rabbit mAb (Cell Signaling Technology, cat. #14958) or IgG Rabbit mAb (Cell Signaling Technology, cat. #3900) control. The immunoprecipitated DNA and input DNA were purified and amplified by qRT‐PCR with primers as follows: *ACSL4*: forward primer, 5′‐CAGGCATGCTTACTGTCCAG‐3′; reverse primer, 5′‐TGAGCCTGCAAAACTTCCTG‐3′.

### 2.11 mRNA stability assay

C4‐2 or PC3 VEC and CDK12^KO^ cells incubated with complete RPMI‐1640 were treated with 10 μg/mL actinomycin D for 0, 3 or 6 h. No decrease in cell viability was observed during the experiment. Total RNA was collected with RNeasy mini kits, and qRT‐PCR analysed mRNA levels. All samples were normalized with ACTB. The ACSL4 expression level was detected using primer *ACSL4*‐F (5′‐CATCCCTGGAGCAGATACTCT‐3′), *ACSL4*‐R (5′‐ TCACTTAGGATTTCCCTGGTCC‐3′). The *ACSL4*‐E1 expression level was detected using primer *ACSL4*‐E1‐F (5′‐AGAGAAAAAGAGGACATTTGCGT‐3′), *ACSL4*‐E1‐R (5′‐AGCCAAGGCAGTTCAATCTTAGT‐3′).

### 2.12 ATP level test

ATP levels were measured using the ATP bioluminescence detection kit (Beyotime, cat. #S0026). Briefly, 1 × 10^5^ cells were seeded in 6‐well plates for each well, after 24 h, treated with 10 μM rotenone or DMSO for 8 h, cells were collected and washed with PBS and pyrolyzed with a lysis buffer supplied with the kit. The homogenate was centrifuged at 12 000 × *g* for 5 min at 4°C. The supernatant was collected for ATP detection. The protein concentration of the supernatant was measured using the Bradford reagent. Furthermore, 100 μL of supernatant and 100 μL of ATP detection buffer were mixed, and the luminescence was measured using a microplate reader (BioTek Co.). Gradient dilution of the standard solution was conducted to generate the standard curve. ATP levels were calculated according to the standard curve and normalized against the standards’ protein concentration.

### 2.13 Mitochondrial membrane potential

According to the manufacturer's instructions, 1 × 10^6^ C4‐2 or PC3 VEC and CDK12^KO^ cells were seeded into 6‐well plates in conditional media, and after 24 h, treated cells with DMSO or 10 μM rotenone for 8 h. Then, mitochondrial membrane potential was measured by the JC‐1 kit (Beyotime, cat. #C2006) using LSM 980 confocal microscopy (Zeiss Co.). Image quantification was done using ImageJ.

### 2.14 Intracellular ROS measurement

According to the manufacturer's instructions, intracellular ROS production was determined by staining cells with 10 μM CM‐H2DCFDA (Invitrogen, cat. #C6827). 1 × 10^6^ cells were seeded in 6‐well plates, after 24 h, treated with 10 μM rotenone or DMSO for 8 h. Then, stained cells were analysed by flow cytometers (Agilent Co.).

### 2.15 Apoptosis measurement

According to the manufacturer's instructions, 1 × 10^6^ C4‐2 or PC3 VEC and CDK12^KO^ cells were seeded in 6‐well plates, after 24 h, treated cells with DMSO or 10 μM rotenone for 8 h. Then, apoptosis was calculated using the Apoptosis Detection Kit (BD Biosciences, cat. #556547). Briefly, cells were simultaneously stained with Annexin V‐fluorescein isothiocyanate and propidium iodide (PI) for 30 min at room temperature. Apoptosis was examined by flow cytometry (Agilent Co.).

### 2.16 Xenograft mice model

Nude (nu/nu) mice were bought from the Gempharmatech Co.. 2.5 × 10^6^ of PC3 VEC or PC3 CDK12^KO^ cells were inoculated subcutaneously into 6‐week‐old male nude mice. 7.5 mg/kg IACS‐010759 (Selleck, cat. #S8731) and the vehicle (.5% methylcellulose [Selleck, cat. #E2934] and 4% DMSO) were administered orally once every other day. Five mice were used in each group. Body weight and tumour volume are monitored every other day. Tumour volume (mm^3^) = (length × width^2^)/2.

### 2.17 Bioinformatics analysis

All sequencing data are from our RNA‐seq data, metabolome data, cbioportal (cbioportal.org) database and GEO (ncbi.nlm.nih.gov/geo/) database. The principal component analysis (PCA) and enrichment of metabolite pathways were performed by MetaboAnalyst online software (metaboanalyst.ca), with the enrichment analysis performed using the package global test. For RNA‐seq data analysis, clean sequencing reads were aligned to hg38 reference assembly (Ensembl Assembly 93) with star 2.6.0a (GPL v3, open source), and a > 95% alignment rate was achieved for each sample. Differential expression analyses were performed with the DESeq2 R package. For each type of tissue, genes with total counts less than 50 summarized for all six samples were filtered out first. Genes were considered differentially expressed when they had a | log2(fold change) | > log22 and *p*‐value < .05. For Gene Set Enrichment Analysis (GSEA), a ranked list was generated according to the log fold change (logFC) for DEGs between all CDK12^KO^ cases and controls. GSEA was performed using the clusterProfiler R package. The enrichment score (ES) was derived by calculating the weighted Kolmogorov–Smirnov statistic to a running sum of the ranked list. The ES was further normalized to account for the size of each gene set. False discovery rates (FDRs) less than .05 were considered statistically significant.

### 2.18 Statistical analysis

All experiments were performed thrice or more, and data were presented as the mean ± standard deviation (s.d.). Statistically significant differences between the two groups were assessed using an unpaired *t*‐test. *p* < .05 was considered significant. Statistically significant differences for overall survival analysis were found using the Log‐rand (Mantel−Cox) test, and for gene expression correlation, the Pearson *r* correlation analysis was used. All statistical analyses were performed using GraphPad Prism software and Microsoft Excel.

## 3 RESULTS

### 3.1 CDK12‐deficient prostate cancer is associated with a worse prognosis

**FIGURE 1:**
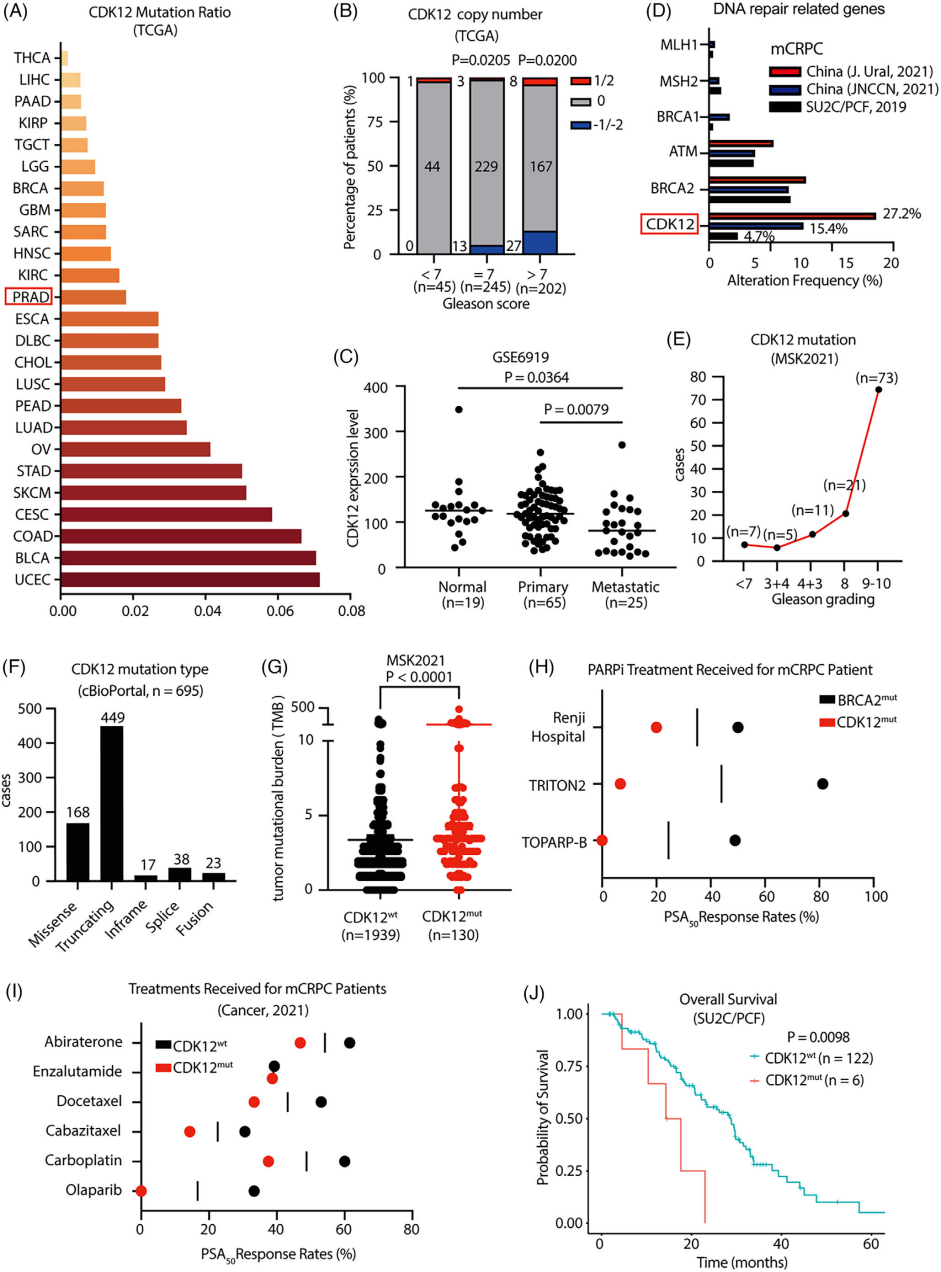
CDK12‐deficient prostate cancer is associated with a worse prognosis. (A) Mutation frequency of CDK12 in different cancers. Data were obtained from the cBioPortal database (cbioportal.org). (B) As patients’ Gleason scores increased, CDK12 gene copy number loss rose. 1/2 means CDK12 gain; 0 means CDK12 normal; −1/−2 means CDK12 loss. Data were subjected to a one‐way ANOVA test. (C) CDK12 expression was significantly lower in patients with metastatic prostate cancer than in normal people and early‐stage prostate cancer patients. Data were shown as median and subjected to an unpaired *t*‐test. (D) The mutation frequency of CDK12 in Chinese prostate cancer patients is significantly higher than the world average and other DNA damage repair‐related genes. For SU2C/PCF Dream Team (2019) sequencing data, *n* = 444. The Chinese patient data were obtained from Renji Hospital, Shanghai, China,^26^, ^33^ *n* = 396 and 292. (E) As the Gleason grade of prostate cancer patients increases, the frequency of CDK12 mutations also rises. (F) The mutated forms of CDK12 in prostate cancer patients. (G) Prostate cancer patients with CDK12 mutations have a significantly higher TMB than patients without CDK12 mutations. Data were shown as means ± s.d. and subjected to an unpaired *t*‐test. (H) The PSA_50_ response rate to PARPi was lower in CDK12 mutant patients than in BRCA2 mutant patients. (I) Patients with CDK12 mutations had lower PSA_50_ response rates to different drugs than patients harbouring mutations in other DNA damage repair genes (BRCA2 and ATM).^27^ (J) Overall survival rate of prostate cancer patients with CDK12 mutations was significantly lower than that of CDK12 wild‐type patients. Data were subjected to the Log‐rank (Mantel−Cox) test.

CDK12 mutation is commonly found in various cancers (Figure 1A). CDK12‐mutated prostate cancer has been classified as a unique subtype of prostate cancer.^19^ We analysed sequencing data from patient samples in public databases and found a higher CDK12 copy number loss frequency in patients with higher Gleason scores (Figure 1B). The expression of CDK12 was lower in prostate cancer samples compared to normal prostate tissue, and CDK12 levels were further significantly lower in metastatic patients than in early‐stage patients (Figure 1C), suggestive of its tumour suppressive role in prostate cancer progression. The incidence of prostate cancer varies by region and ethnicity, with the highest incidence among Caribbean males and the lowest among East Asian males.^32^ Surprisingly, CDK12 mutation frequency in prostate cancer showed a significantly higher rate in Chinese patients,^26^, ^33^ with 15.4%−27.2% compared to approximately 4.7% among global prostate cancer patients (Figure 1D). Furthermore, CDK12 mutation frequency substantially increased with an elevated Gleason score (Figure 1E), and truncating mutations are the most common form of CDK12 mutation (Figure 1F), leading to the loss of CDK12 function. CDK12‐mutated prostate cancer patients also had a higher tumour mutation burden (TMB) than CDK12 wild‐type prostate cancer patients (Figure 1G), which leads to an increased generation of neoantigens that may result in a better response to immune therapies.^19^

CDK12 is considered as a DNA repair‐related gene because it phosphorylates RNA Pol II to regulate the expression of HR genes (such as BRCA2 and ATM).^15^, ^16^ Recently, PARPi has been approved to treat BRCA1/2‐and ATM‐mutated CRPC.^34^ However, from clinical data, the response rate of CDK12‐mutated prostate cancer patients to PARPi was much lower than that of patients with BRCA2 mutations (Figure 1H). In addition, a multicentre study also showed that prostate cancer patients with CDK12 mutations had lower PSA_50_ responses to androgen receptor pathway inhibitors (abiraterone or enzalutamide) and chemotherapy drugs than patients with other DDR gene defects (Figure 1I), suggestive of a worse prognosis of CDK12‐mutated prostate cancer patients. As a result, prostate cancer patients with CDK12 mutations have significantly shorter overall survival than patients without mutations (Figure 1J). Therefore, the worse prognosis observed in CDK12‐deficient CRPC underscores the imperative for the underlying mechanism studies and the alternative treatment development.

### 3.2 CDK12 deficiency reprogrammes cellular energy metabolism to cause a ferroptosis potential

**FIGURE 2:**
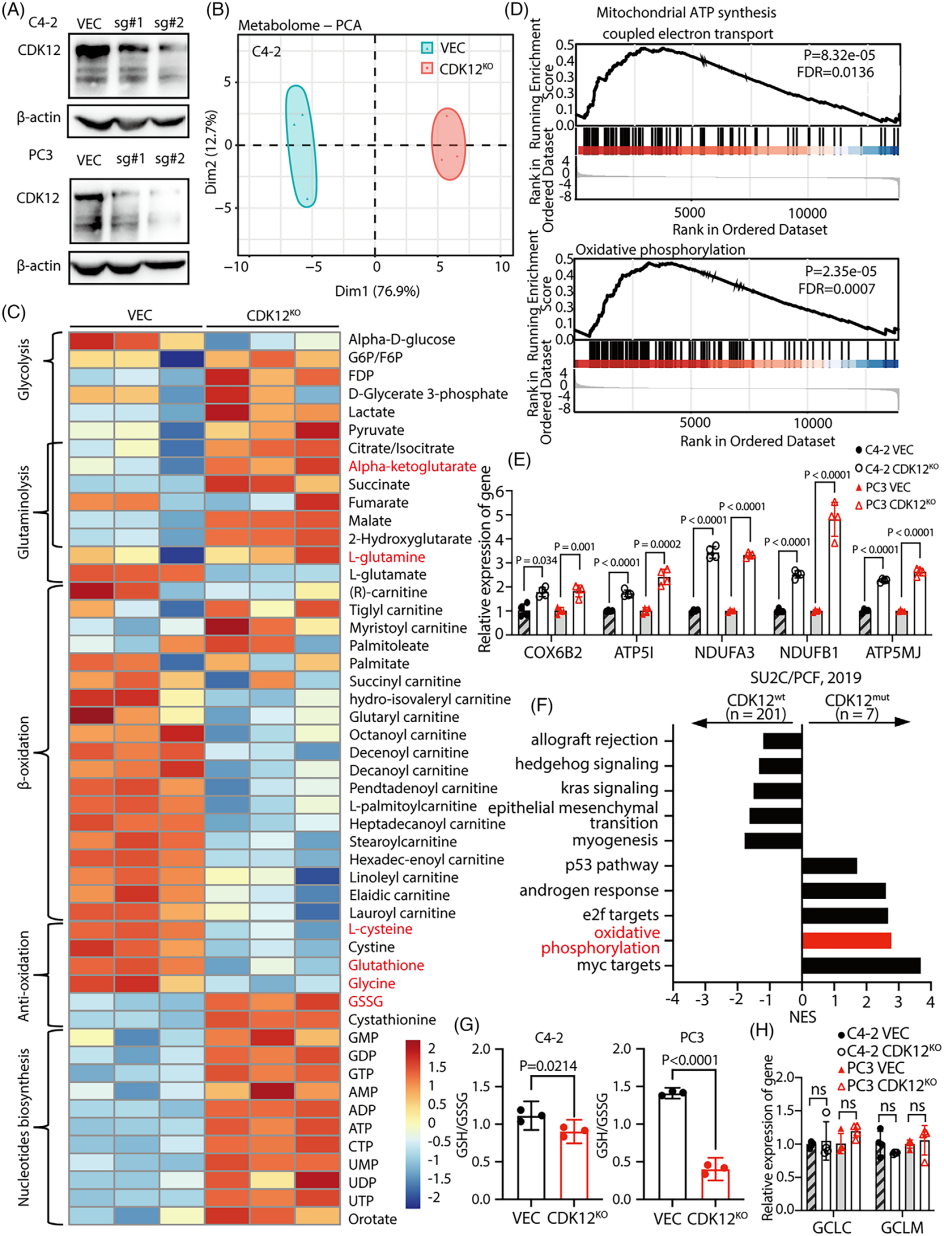
CDK12 deficiency reprogrammes cellular energy metabolism, causing ferroptosis potential. (A) Knockout of CDK12 in CRPC cell lines C4‐2 and PC3 using CRISPR‐Cas9 technology to obtain stably transformed cell lines. (B) PCA analysis of differentially expressed metabolites revealed that CDK12 knockout affects the metabolic profiles of C4‐2 cells. (C) Heatmap of metabolite levels were altered in C4‐2 cells with CDK12 knockout. (D) GSEA pathway enrichment analysis showed that mitochondrial ETC and oxidative phosphorylation‐related genes were enriched in highly expressed regions in C4‐2 cells after CDK12 knockout. Data were subjected to a Student's *t*‐test and Benjamini−Hochberg correction. (E) The expression levels of mitochondrial ETC and oxidative phosphorylation‐related genes were detected using qRT‐PCR (*n* = 4 parallel repeat). The experiments were repeated in three biologically independent cell experiments. (F) GSEA pathway enrichment analysis showed that oxidative phosphorylation‐related genes were enriched in the CDK12 mutation patients’ group. Data were subjected to a Student's *t*‐test and Benjamini−Hochberg correction. (G) The GSH/GSSG ratio was significantly decreased in C4‐2 and PC3 cells with CDK12 knockout (*n* = 3 biologically independent repeat). (H) The *GCLC* and *GCLM* expression levels were detected using qRT‐PCR (*n* = 4 parallel repeat). The experiments were repeated in three biologically independent cell experiments. Data were shown as means ± s.d. and subjected to an unpaired *t*‐test, ns means *p* > .05.

Tumour progression is accompanied by tumour metabolism alterations and, vice versa, tumour metabolism reprogramming contributes to tumour progression and therapy resistance.^2^, ^3^ To examine the effects of CDK12 deficiency on the cellular metabolism of CRPC cells, we used CRISPR‐Cas9 technology to establish CDK12 knockout CRPC cell lines (Figure 2A). We did a metabolite profiling for C4‐2 cells with or without CDK12 knockout. PCA clearly showed that CDK12 knockout significantly reprogrammed the overall metabolite profiles (Figure 2B). Further metabolite heatmap analysis revealed that CDK12 knockout led to a significant increase in metabolic products related to glycolysis, glutaminolysis and nucleotide biosynthesis, while a substantial decrease in metabolites involved in fatty acid beta‐oxidation (Figure 2C), indicating that CDK12 deficiency significantly alters the energy metabolism of tumour cells. Of particular interest is that almost all tricarboxylic acid (TCA) cycle intermediate metabolites, including pyruvate, citrate/isocitrate, alpha‐ketoglutarate, succinate and malate, were significantly upregulated in CDK12 knockout cells (Figure 2C), suggesting that mitochondrial function is enhanced in CDK12‐deficient cells to supply energy for cell proliferation and growth.

To further investigate the mechanism by which CDK12 regulates tumour metabolism, we conducted RNA sequencing on CDK12 control and knockout C4‐2 cells. GSEA pathway enrichment analysis revealed that the genes related to the mitochondrial ETC and oxidative phosphorylation were primarily enriched in the highly expressed group in CDK12 knockout cells (Figure 2D). We validated the expression levels of the top five genes with the most significant expression changes among all ETC and oxidative phosphorylation‐related genes in our RNA‐seq data using qRT‐PCR. The results indicated a significant upregulation of these genes in CDK12 knockout C4‐2 and PC3 cells (Figure 2E and Figure S1A). Patients’ transcriptomic data analysis from SU2C/PCF also revealed that the expression of those genes was negatively correlated with CDK12 (Figure S1B–F). Additionally, CRPC patient data dependent‐GSEA pathway analysis indicated an increased oxidative phosphorylation in the CDK12‐mutated patient group (Figure 2F), consistent with our cellular RNA sequencing data. All these findings support the idea that enhanced mitochondrial functions were found in CDK12 knockout CRPC cells with more active electron transport activity.

It has been known that ATP production in mitochondria comes with ROS.^9^, ^30^ The more active ETC activity in CDK12 knockout cells may suggest that CDK12 regulates the cellular redox status. Indeed, we observed a decrease in cysteine and GSH levels but a significant increase in GSSG levels in CDK12 knockout cells (Figure 2C), suggesting a more oxidative status in CDK12 knockout cells. We measured the ratio of GSH to GSSG in cells and found a significant decrease in CDK12 knockout C4‐2 and PC3 cells (Figure 2H). At the same time, the GSH/GSSG synthesis gene did not have substantial changes in expression (Figure 2H), indicating a rapid depletion of GSH and decreased intracellular antioxidant capacity, which may suggest a potential lipid ROS‐induced ferroptosis susceptibility for CDK12 knockout cells (Figure S2A).

### 3.3 CDK12‐deficient CRPC cells escape ferroptosis by downregulating ACSL4 expression

**FIGURE 3:**
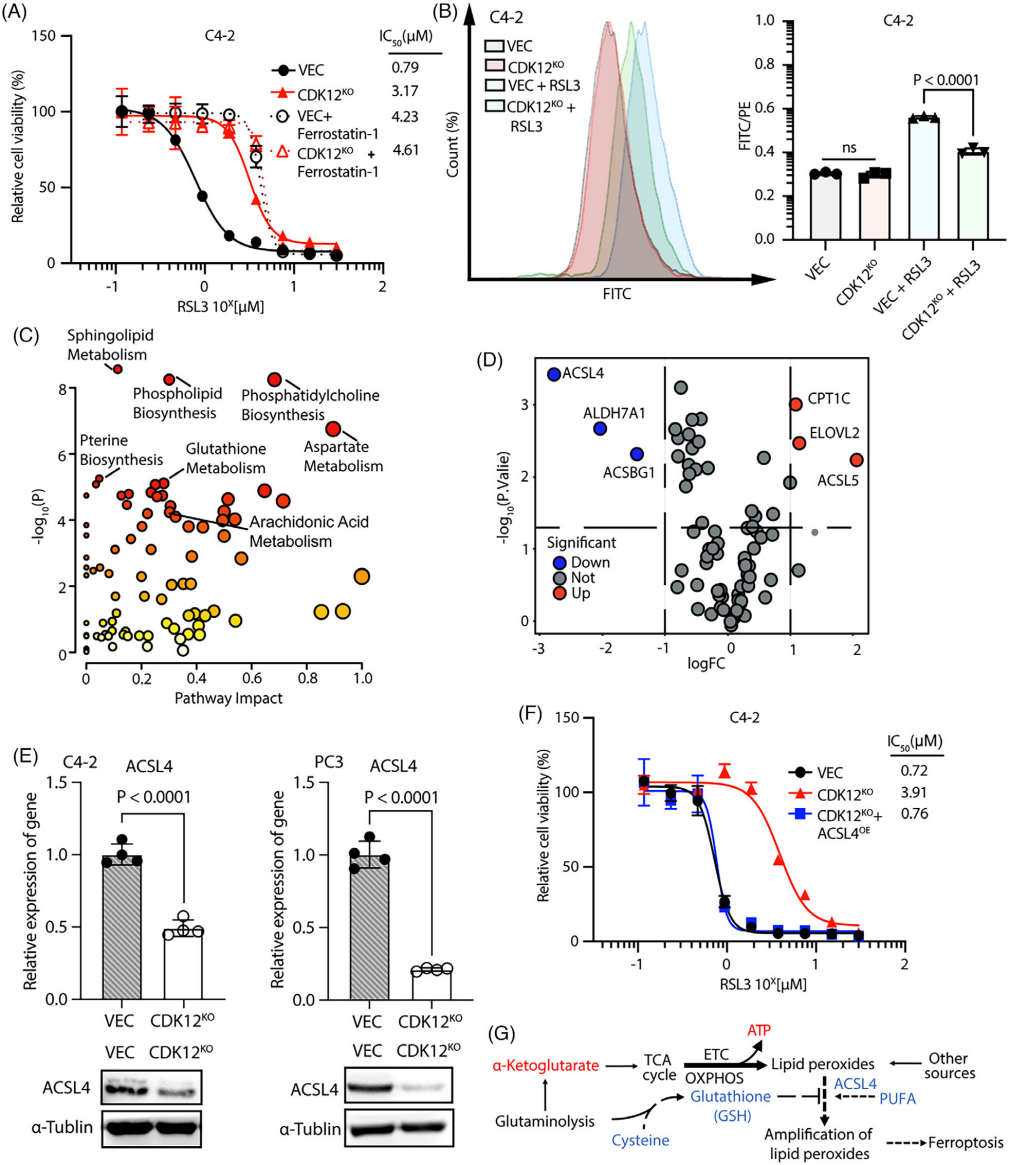
CDK12‐deficient CRPC cells escape ferroptosis by downregulating ACSL4 expression. (A) Treating C4‐2 VEC cells and C4‐2 CDK12^KO^ cells with RSL3 with or without ferrostatin‐1 (2 μM) for 48 h using a series of concentrations (*n* = 3 biologically independent repeat). The chemiluminescence assay measured the cell count, and the IC_50_ value was calculated using GraphPad Prism10. (B) Membrane lipid ROS levels were lower in CDK12^KO^ C4‐2 cells after treatment with RSL3. They were treated cells with 2 μM RSL3 or DMSO for 12 h. Cell membrane ROS levels were measured using the BODIPY‐C11 probe, and the fluorescence signals were analysed and quantified using flow cytometry (*n* = 3 biologically independent repeat). (C) Analysis of differentially expressed metabolite enrichment pathways in C4‐2 cells with CDK12 knockout. Data were analysed using Metaboanalyst. Data were subjected to a Student's *t*‐test. (D) Lipid metabolism‐related differentially expressed genes in C4‐2 CDK12 knockout cells. Data were subjected to a Student's *t*‐test. (E) CDK12 knockout significantly decreased mRNA and protein expression of ACSL4 in CRPC C4‐2 and PC3 cells (*n* = 4 parallel repeat). The experiments were repeated in three biologically independent cell experiments. (F) Overexpression of ACSL4 restored the sensitivity of CDK12 knockout C4‐2 cells to ferroptosis inducers (*n* = 3 biologically independent repeat). (G) CDK12‐deficient CRPC cells counterbalance ferroptosis stress caused by active ETCs by inhibiting ACSL4 expression. Data were shown as means ± s.d. and subjected to an unpaired *t*‐test, ns means *p* > .05.

To our surprise, CDK12 knockout CRPC C4‐2 and PC3 cells showed a significant resistance to ferroptosis induced by RSL3 (inhibitor of GPX4) or Erastin (inhibitor of SLC7A11)^35^ compared to CDK12 control cells (Figure 3A and Figure S2B−D), with *GPX4* and *SLC7A11* did not have significant changes in mRNA level (Figure S2E). Treatment with the ferroptosis inhibitor ferrostatin‐1 (a membrane radical scavenger)^35^ remarkably rescued the cell death induced by RSL3 or Erastin, confirming that the death‐inducing effect of these two drugs is mediated by ferroptosis (Figure 3A and Figure S2B–D). To directly detect the occurrence of ferroptosis, we also used BODIPY‐C11 probes to measure the levels of lipid ROS on the cell membrane. We found that the lipid ROS level on the cell membrane of CDK12 knockout CRPC cells was significantly lower than that of CDK12 control cells after treatment with ferroptosis inducers (Figure 3B and Figure S1E), indicating that CDK12 does regulate RSL3 and Erastin‐induced ferroptosis.

As ferroptosis is caused by membrane lipid peroxidation, we analysed the metabolic pathways of altered metabolites in control and CDK12 knockout cells. We found that lipid metabolisms, including the phospholipid biosynthesis pathway, were among the most enriched pathways altered in CDK12 knockout cells (Figure 3C), implying that CDK12 modulates lipid metabolism that may be related to ferroptosis. On the other hand, we analysed the expression levels of all lipid metabolism‐related genes from our RNA‐seq dataset. *CPT1C*, *ELOVL2* and *ACSL5* expression levels significantly increased after CDK12 knockout. In contrast, the expression levels of *ACSL4*, *ALDH7A1* and *ACSBG1* showed a significant decrease (Figure 3D). The ACSL4 expression was the most significant downregulated in CDK12 knockout cells, and it has been well‐known that ACSL4 plays an essential role in regulating ferroptosis.^36^, ^37^

We then validated the downregulation of ACSL4 at both transcriptional and protein levels in C4‐2 and PC3 cells, respectively (Figure 3E). To provide direct evidence that the downregulation of ACSL4 mediates the resistance of cells to ferroptosis caused by CDK12 deficiency, we overexpressed ACSL4 in CDK12 knockout CRPC cells and, as expected, the recovery of ACSL4 expression restored the sensitivity of cells to ferroptosis inducers (Figure 3F and Figure S2G,H). These data demonstrate that CDK12 deficiency enables CRPC cells to escape ferroptosis by downregulating ACSL4 expression despite its deficiency causing a high ferroptosis potential in cells (Figure 3G).

### 3.4 The downregulation of ACSL4 by CDK12 is dependent on its kinase activity

**FIGURE 4:**
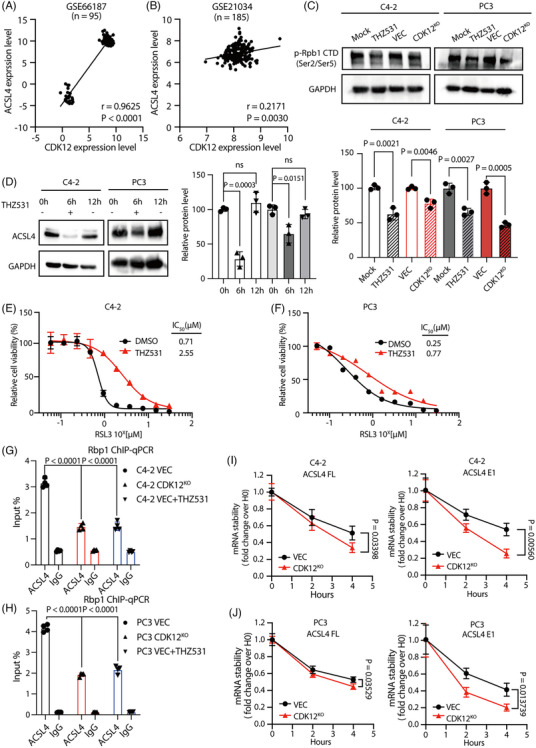
The downregulation of ACSL4 expression by CDK12 is dependent on its kinase activity. (A, B) A positive correlation between *ACSL4* and *CDK12* mRNA expression in CRPC patients. Data were analysed using Pearson correlation analysis. (C) CDK12 knockout and THZ531 decreased the overall phosphorylation level of RNAPol II in CRPC cells. Cells were treated with 1 μM THZ531 for 12 h. The experiments were repeated in three biologically independent cell experiments. (D) Inhibition of CDK12 kinase activity by THZ531 effectively reduced ACSL4 expression in CRPC cells, and withdrawal of THZ531 restored ACSL4 expression. The experiments were repeated in three biologically independent cell experiments. (E, F) Inhibition of CDK12 kinase activity by THZ531 renders CRPC cells resistant to ferroptosis inducer RSL3. They treated cells with RSL3 in the presence or absence of 1 μM THZ531 for 48 h (*n* = 3 biologically independent repeat). (G, H) qRT‐PCR analysis of each ChIP‐DNA sample was performed for *ACSL4* (*n* = 4 parallel repeat). The experiments were repeated in three biologically independent cell experiments. Results are reported as fold enrichment of immunoprecipitated DNA from each sample relative to the DNA immunoprecipitated with the non‐specific antibody IgG. All samples were pre‐treated for 1 h with 10 μg/mL actinomycin D. (I, J) CDK12 knockdown reduced the stability of *ACSL4* nascent mRNA and full‐length mRNA in CRPC cells. Cells were treated with 10 μg/mL actinomycin D to inhibit cellular transcription, after which mRNA levels were detected by qRT‐PCR (*n* = 4 parallel repeat). The experiments were repeated in three biologically independent cell experiments. Data were shown as means ± s.d. and subjected to an unpaired *t*‐test, ns means *p* > .05.

To investigate how CDK12 regulates ACSL4 expression, we first analysed the correlation between CDK12 and ACSL4 with the CRPC patient database and found a significant positive correlation between *CDK12* and *ACSL4* mRNA expression levels (Figure 4A,B). Together with our RNA‐seq data (Figure S3A) and qRT‐PCR validation (Figure 3E), these results suggest that CDK12 may regulate the expression of ACSL4 at the mRNA level.

CDK12 regulates gene expression mainly by phosphorylating RNA Pol II CTD, controlling transcription elongation and splicing.^12^, ^13^, ^14^, ^15^, ^16^ As expected, the overall phosphorylation level of RNA Pol II in prostate cancer cells was significantly decreased with CDK12 knockout (Figure 4C). To determine whether the regulation of ACSL4 expression by CDK12 depends on its kinase activity, we treated the cells with the CDK12 kinase activity inhibitor THZ531, which can inhibit the kinase function of CDK12, and lead to a reduction in the overall phosphorylation level of RNA Pol II CTD Ser2 & Ser5 in the cells (Figure 4C). Remarkably, after treating cells with THZ531 for 6 h, a significant decrease in the expression level of *ACSL4* was observed (Figure 4D). Interestingly, upon the removal of THZ531, the *ACSL4* expression level returned to the average (Figure 4D). Meanwhile, using THZ531 to inhibit CDK12 kinase activity also induced the resistance of prostate cancer cells to ferroptosis inducers (Figure 4E,F). Similar phenomena of ferroptosis resistance have been observed in colon cancer cell line HCT116, lung cancer cell line A549 and melanoma cell line A375 (Figure S3B–D), indicating that the ferroptosis regulation of CDK12 is dependent on its kinase activity.

To demonstrate that CDK12 directly affects the phosphorylation of RNA Pol II CTD and thereby influences the transcriptional selection of ACSL4 by RNA Pol II, we performed ChIP assays for RNA Pol II in CDK12 knockout, control and THZ531‐treated CRPC cells. Subsequently, qRT‐PCR analysis using primers designed from the ACSL4's exon6 sequence (Figure S3E) revealed a significant decrease in RNA Pol II binding to ACSL4 upon CDK12 knockout or inhibition of its kinase activity (Figure 4G,H), providing direct evidence of the vital role of CDK12 kinase activity in maintaining ACSL4 transcription. Further, to examine whether CDK12 deficiency affects ACSL4 mRNA degradation rate, we designed qRT‐PCR primers within the first exon of ACSL4 to reflect the stability of ACSL4's nascent mRNA and treated cells with actinomycin D to inhibit transcription. As expected, CDK12 knockout significantly increased the degradation rate of both ACSL4's nascent mRNA and full‐length mRNA in prostate cancer cells (Figure 4I,J), indicating that CDK12 deficiency downregulates ACSL4 expression levels by influencing the transcriptional selection of ACSL4 by RNA Pol II and decreasing the stability of ACSL4 mRNA.

### 3.5 CDK12‐deficient CRPC exhibits a heightened dependence on ETC

**FIGURE 5:**
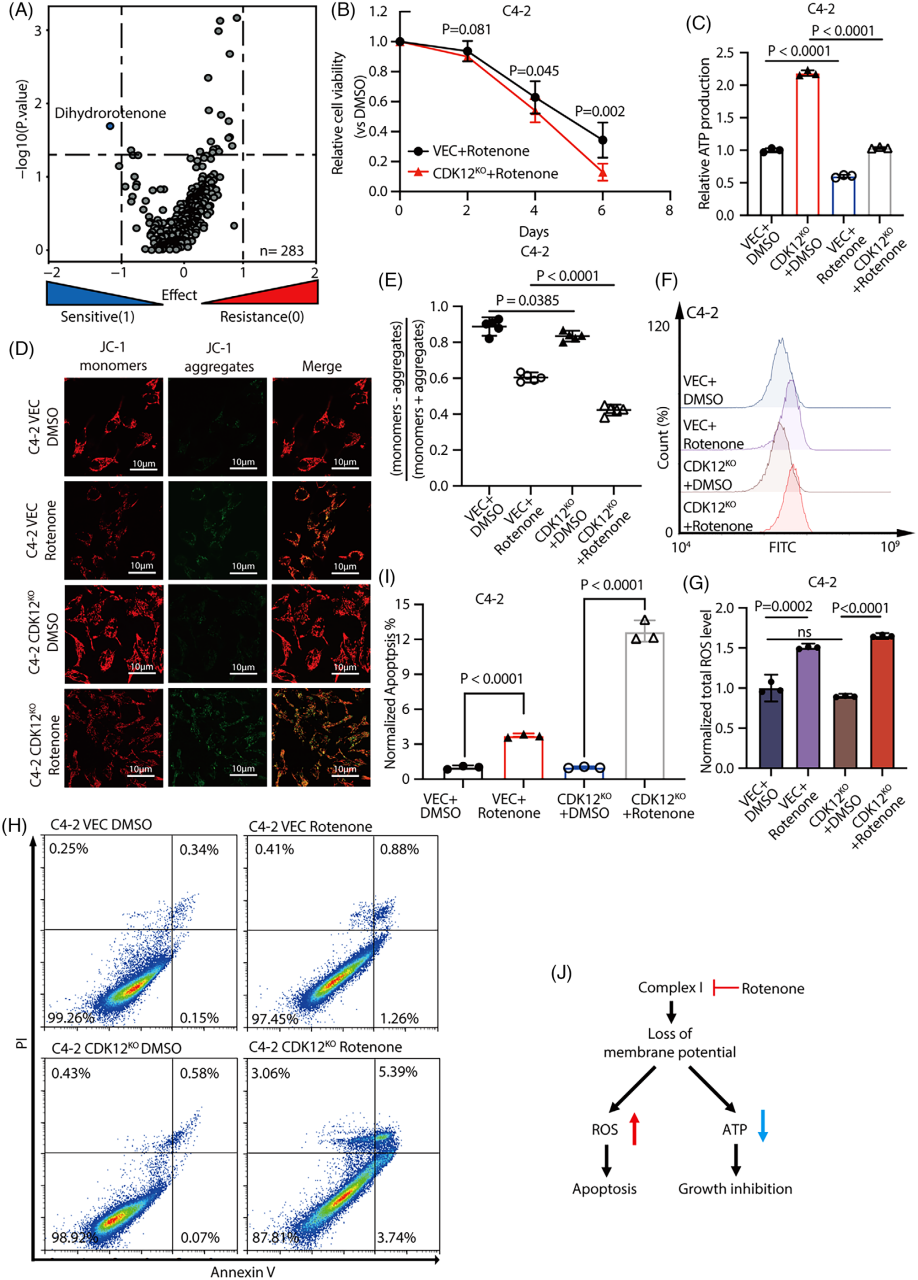
CDK12‐deficient CRPC exhibits a heightened dependence on the functionality of ETC and is more sensitive to ETC inhibitors. (A) Cancer cell lines with CDK12 mutations are more sensitive to dihydrorotenone, according to data from the Genomics of Drug Sensitivity in Cancer database (cancerrxgene.org). Data were subjected to a Student's *t*‐test. (B) Rotenone inhibited the growth of C4‐2 cells effectively. Cells were treated with 1 μM rotenone for 6 consecutive days (*n* = 3 biologically independent repeat). (C) An increased ATP level in C4‐2 CDK12 knockout cells. Rotenone can inhibit the production of ATP in CRPC cells, and it is more effective in cells with CDK12 knockout (*n* = 3 biologically independent repeat). (D, E) Rotenone can disrupt the mitochondrial membrane potential of C4‐2 cells, and this disruption is more pronounced in cells with CDK12 knockout. The mitochondrial membrane potential was characterized using the JC‐1 probe and observed using confocal microscopy. Monomers represent average mitochondrial membrane potential, while aggregates represent the loss of mitochondrial membrane potential (*n *= 3 biologically independent repeat). Quantifying fluorescent signals using image J. (F, G) Rotenone caused elevated total ROS levels in C4‐2 cells. Total ROS levels were detected using the CM‐H2DCFDA probe, and the fluorescence signals were analysed and quantified using flow cytometry (*n* = 3 biologically independent repeat). (H, I) CDK12 knockout cells have a higher percentage of apoptosis induced by Rotenone. Flow cytometry was used to separate and quantify the cells (*n* = 3 biologically independent repeat). (J) Inhibition of ETC complex I inhibits ATP synthesis and elevates cellular ROS levels in CRPC cells, inducing apoptosis. This effect was more pronounced in CDK12‐deficient cells. Data were shown as means ± s.d. and subjected to an unpaired *t*‐test, ns means *p* > .05.

To seek more efficient treatment drugs for CDK12‐deficient prostate cancer, we examined the response of pan‐cancer cell lines with CDK12 mutations to different medications in the Genomics of Drug Sensitivity in Cancer database. Amazingly, CDK12‐mutated cancer cell lines are most sensitive to dihydro‐rotenone, a known inhibitor of mitochondrial ETC complex I and the only one among nearly 300 tested compounds that shows significance (Figure 5A). This strongly suggests a connection between CDK12 mutations and the mitochondrial ETC.

To confirm this, we treated the cells with rotenone, an analogue of dihydrorotenone with similar functions to dihydrorotenone. As expected, the growth inhibition of prostate cancer cells caused by rotenone significantly increased with CDK12 knockout (Figure 5B and Figure S4A). It is well known that the energy stored in different cellular substances enters the mitochondrial respiration chain via other metabolic pathways and is utilized for ATP synthesis by transferring electrons (Figure S5A).^38^ We first examined the intracellular levels of ATP and found that more ATP production was observed in CDK12‐deficient prostate cancer cells than in control cells (Figure 5C and Figure S4B). Furthermore, rotenone treatment resulted in a more significant decrease in ATP levels in CDK12‐knockout cells than in control cells (Figure 5C and Figure S4B). Next, we asked whether the ATP synthesis in CDK12‐deficient CRPC cells depends on the supply of a particular energy source. We treated cells with 2DG (HK inhibitor), CB839 (GLS1 inhibitor) and Etomoxir (CPT1 inhibitor) to inhibit the catabolism of glucose, glutamine and fatty acids, respectively, blocking the supply of each of these three primary energy sources to cellular ATP synthesis. Surprisingly, CDK12 knockout CRPC cells were not more sensitive to any of these three compounds than control cells (Figure S5B–D), reflecting the dependence of CDK12‐deficiency CRPC cells on mitochondrial ETC energy supply. This also agrees with the metabolite profiling results that CDK12 knockout altered glycolysis, glutaminolysis and fatty acid beta‐oxidation (Figure 2C).

To further investigate the mechanism by which rotenone induces cell death in CRPC cells, we employed the JC‐1 probe to characterize the mitochondrial membrane potential. We found that rotenone treatment resulted in a partial loss of mitochondrial membrane potential, and this phenomenon was more pronounced in CDK12 knockout CRPC cells (Figure 5D,E and Figure S4C,D), possibly due to the increased activity of the ETC caused by CDK12 knockout. Loss of the mitochondrial membrane potential usually leads to increased intracellular ROS levels.^9^, ^30^ We examined the overall cellular ROS level changes before and after rotenone treatment to verify this. A more significant increase in total ROS levels was observed in CDK12 knockout CRPC cells compared to control cells after treatment with rotenone (Figure 5F,G and Figure S4E,F). We then examined the effect of rotenone‐induced ROS on cell death. We stained the cells with PI and Annexin X and quantified the proportion of apoptotic cells using flow cytometry. As expected, after the rotenone treatment, the ratio of apoptotic cells was significantly higher in CDK12 knockout cells compared to control cells (Figure 5H,I and Figure S4G,H). These results suggest that rotenone can induce apoptosis by inhibiting the activity of mitochondrial complex I, and this inhibitory effect on cancer cells is more pronounced in CDK12‐deficient CRPC cells (Figure 5J).

### 3.6 ETC inhibitors suppress CDK12‐deficient CRPC tumours in vitro and in vivo

**FIGURE 6:**
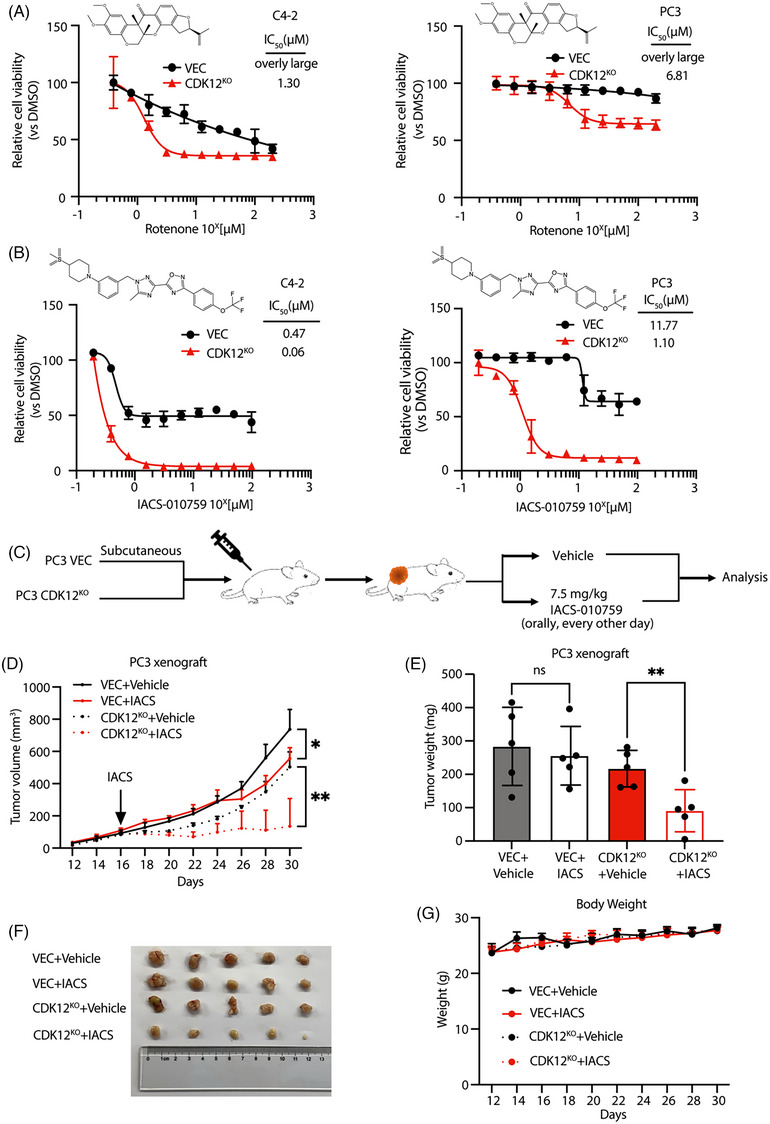
Mitochondrial ETC inhibitors suppress mCRPC tumours in vivo and in vitro. (A) CDK12 knockout CRPC cells are more sensitive to Rotenone (*n* = 3 biologically independent repeat). (B) CDK12 knockout CRPC cells are more sensitive to IACS‐010759 (*n* = 3 biologically independent repeat). (C) Schematic representation of in vivo validation of the therapeutic effect of IACS‐010759. (D) The volume of CDK12 control and deficient CRPC xenograft treated with or without IACS‐010759 (*n* = 5 biologically independent animals). (E) Changes in tumour weight of CDK12 wild‐type and CDK12‐deficient CRPC after treatment with IACS‐010759. IACS‐010759 has a better therapeutic effect on CDK12‐deficient CRPC (*n* = 5 biologically independent animals). (G) Body weights of tumour‐bearing mice (*n* = 5 biologically independent animals). Data were shown as means ± s.d. and subjected to an unpaired *t*‐test, ns means *p* > .05; *p < .05; **p < .01; ***p < .001.

Several drugs targeting ETC have been used for clinical research in solid tumours and acute myeloid leukaemia (AML).^38^ We tested nine drug compounds that underwent clinical trials for their inhibitory effects on CDK12 control and knockout CRPC cells, including complex I/III/IV/V inhibitors. Most of these drugs did not achieve satisfactory results in C4‐2 and PC3 cell lines (Figure S6A–H). However, compound IACS‐010759 showed a similar inhibitory effect on C4‐2 and PC3 cells compared to Rotenone, and the inhibitory effect on CDK12 knockout cells was significantly more substantial than that on control cells (Figure 6A,B). IACS‐010759 is a small molecule inhibitor of ETC, and it has been shown to have inhibitory effects similar to rotenone while minimizing the toxicity and off‐target effects in the nervous system.^39^, ^40^, ^41^ This drug has shown promising results in phase I clinical trials targeting solid tumours and AML.^40^, ^41^

To further evaluate the inhibitory effect of ETC inhibitors on CRPC cells in vivo, we subcutaneously injected control or CDK12 knockout PC3 cells into male nude mice. We administered IACS‐010759 orally every other day for 2 weeks to the mice when the tumour reached 100 mm^3^ in size and monitored the tumour growth (Figure 6C). Compared to the control xenograft tumours, IACS‐010759 more effectively inhibited the growth of CDK12 knockout xenograft tumours (Figure 6D–F). Additionally, there were no significant changes in the body weight of the mice during the administration period, indicating that the drug did not impact the mice's physical health (Figure 6G). Meanwhile, we also observed that the growth rate of CDK12‐deficient xenograft tumours was slower than that of CDK12 wild‐type xenograft tumours. Similar phenomena have been noticed in recent studies of prostate cancer and several other types of cancer.^42^, ^43^, ^44^, ^45^ However, a large body of clinical evidence has revealed the challenging nature of treating CDK12‐deficient CRPC and its poorer prognosis.^19^, ^26^, ^29^, ^33^, ^46^ Integrating our experimental findings with clinical data, we hypothesize that CDK12 deficiency may not promote the malignant phenotype of prostate cancer by affecting cell proliferation but rather by making tumour cells more resistant to current therapies through metabolic reprogramming and other mechanisms, leading to a worse prognosis. Therefore, the ETC inhibitor IACS‐010759 can effectively inhibit the growth of CRPC tumours in vivo, and it is more effective for CDK12‐deficient CRPC tumours.

## 4 DISCUSSION

**FIGURE 7:**
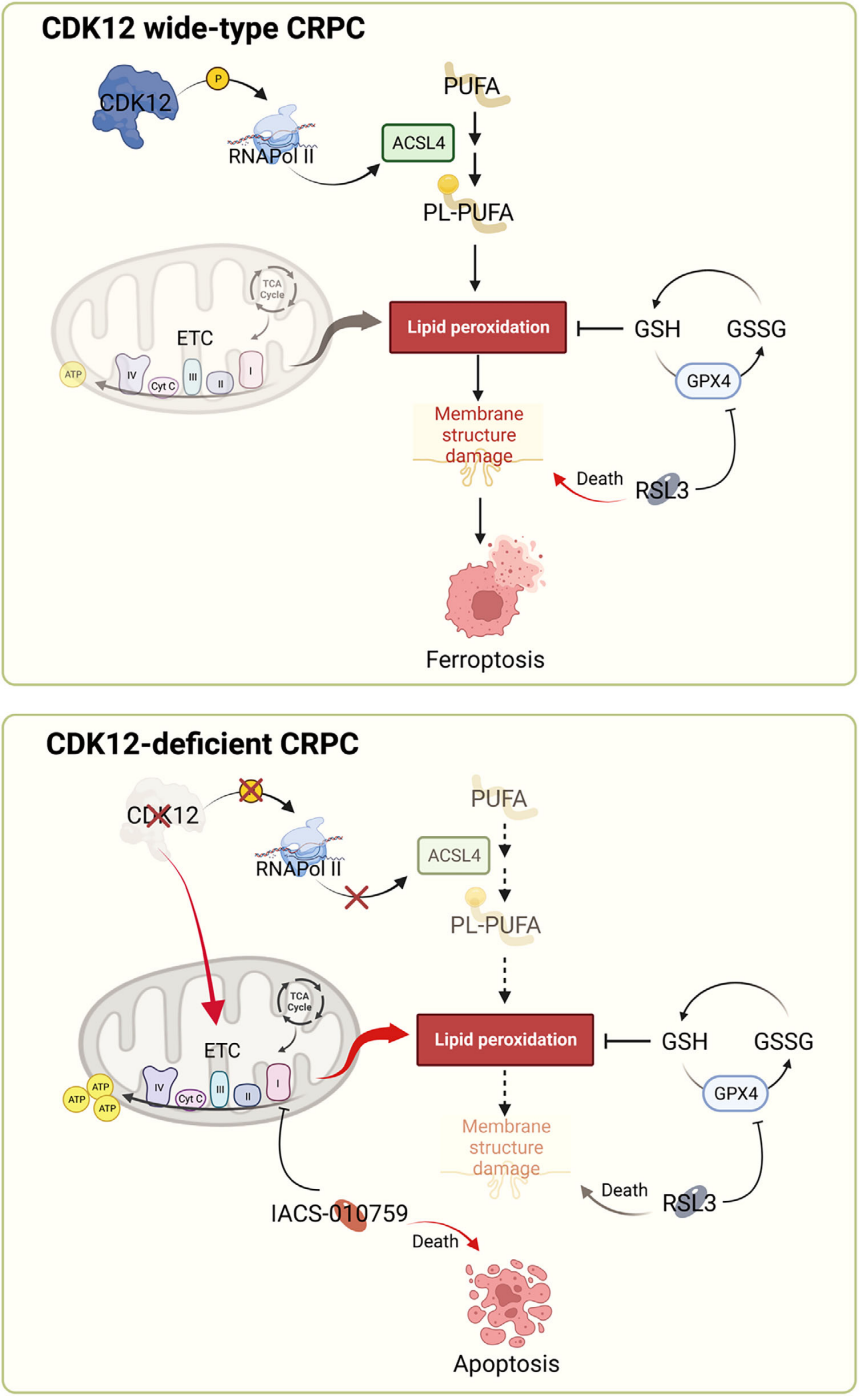
Working model. CDK12 regulates the expression of ACSL4 and ETC‐related genes in CRPC. In CDK12‐deficient CRPC, the expression levels of genes encoding mitochondrial ETC complex components increase, enhancing ETC function. This provides cells with a stable and efficient source of ATP and accelerates the consumption of antioxidant substances such as GSH, increasing the risk of ferroptosis. At the same time, cells reduce the membrane incorporation of PUFA by inhibiting the expression of ACSL4, allowing them to evade ferroptosis and balance the intracellular oxidative stress caused by active ETC function. This is crucial for the survival of cells in a complicated environment in the body and promotes the malignant progression of cancer.

In summary, we have demonstrated that CDK12 deficiency reprogrammes the energy metabolism of CRPC cells, which results in an increased mitochondrial ETC activity to produce more ATP to support CRPC cells’ aggressive growth. This metabolism alteration depletes the intracellular antioxidant substances such as GSH and increases the oxidative stress that endangers the cells at a high risk of ferroptosis. However, CDK12 deficiency downregulates the ACSL4 expression to decrease the incorporation of the ferroptosis substrate PUFA into the membrane and, therefore, offset this potential risk. Meanwhile, CDK12 deficiency‐caused energy metabolism reprogramming may suggest targeting mitochondrial ETC as a promising efficient treatment strategy for CDK12 mutated prostate cancer patients (Figure 7).

CDK12 regulates genes related to HR and impacts genomic stability in prostate cancer.^15^, ^16^, ^47^ Loss‐of‐function mutations in CDK12 can inhibit the transcription of genes like BRCA2 and ATM.^15^, ^16^, ^20^, ^47^ This, in turn, suppresses the DNA damage HR repair pathway and forces the cells to use the non‐homologous end‐joining process to repair damaged DNA.^48^ This increases the likelihood of introducing gene mutations, which promotes tumour development.^19^ However, it also creates opportunities for cancer treatment, like using PARPi to inhibit the HR pathway and kill cancer cells.^49^, ^50^ Additionally, mutations in CDK12 in prostate cancer are often accompanied by extensive tandem duplication, an abnormal form of chromosomal rearrangement.^19^, ^46^ This generates many neoantigens in the cell and may benefit patients in immunotherapy.^19^, ^46^, ^51^ However, PARPi and anti‐PD‐1/L1 therapy still require further clinical validation.^19^, ^25^, ^26^, ^27^, ^46^, ^48^, ^49^, ^50^, ^51^ CDK12‐deficient CRPC continues to be one of the most challenging subtypes of prostate cancer to treat in clinical practice.^19^, ^27^, ^28^, ^29^

It is surprising to note that the frequency of CDK12 mutations in prostate cancer patients varies considerably based on race and geography.^26^, ^33^ While East Asia has a low incidence of prostate cancer, Chinese prostate cancer patients have one of the highest frequencies of CDK12 mutations globally.^26^, ^32^, ^33^, ^52^ This phenomenon could be attributed to various factors and may be related to the androgen metabolism signalling pathway. Since prostate cancer is a hormone‐dependent tumour, the occurrence and progression of the disease are closely linked to the androgen receptor (AR) signalling pathway.^52^ A recent study has suggested that CDK12 can regulate AR's alternative splicing through RNA Pol II phosphorylation, and the loss of CDK12 may lead to resistance of prostate cancer cells to androgen receptor pathway inhibitors (ARSIs).^46^ This also indicates that CDK12 may have a potential regulatory role in the metabolism of tumour cells. CDK12 is capable of promoting breast cancer development through the regulation of one‐carbon metabolism.^53^ However, CDK12's impact on tumour cell metabolism, particularly the overall cellular metabolic landscape, in prostate cancer remains unknown. In this study, we have revealed the metabolic landscape of CRPC with CDK12 deficiency and shown that CDK12 deficiency enhances the energy metabolism of CRPC cells, including improving the TCA cycle and ETC activity, to sustain the growth and survival of tumour cells. Of particular interest is that CDK12 phosphorylation of RNA Pol II is necessary for regular expression of ACSL4 in CRPC cells. In CDK12‐deficient CRPC cells, active electron transport enhances oxidative stress and the risk of ferroptosis. However, cells counteract this risk by inhibiting ACSL4 expression and reducing the incorporation of ferroptosis substrate PUFA into the membrane, thus allowing cells to escape ferroptosis and promote tumour progression.

Energy metabolism is at the core of cellular metabolism, and mitochondria, as the energy factory within the cell, is responsible for almost all energy supply.^5^, ^6^, ^7^ The energy substances the cells uptake are converted into ATP through the mitochondrial ETC, which is used for cell growth, proliferation, movement and other cellular activities.^6^, ^7^, ^9^, ^30^, ^39^ Therefore, targeting the ETC might be a promising efficient cancer therapeutic avenue. However, many mitochondrial respiration inhibitors and oxidative phosphorylation uncouplers, including rotenone, have difficult‐to‐overcome neurotoxicity and widespread off‐target effects and cannot be used for treating human diseases.^54^, ^55^, ^56^, ^57^ Our unbiased screening to search drug compounds for treating CDK12 mutated cancer cells led to identifying dihydro‐rotenone as the most efficient candidate, confirming the connection between ETC and CDK12. Indeed, our study has demonstrated that IACS‐010759, a less toxic ETC inhibitor, has shown promising therapeutic effects on CRPC, especially CDK12‐deficient CRPC cells, in vitro and in vivo, suggesting an efficient therapeutic alternative for CDK12 mutated prostate cancer patients.

## 5 CONCLUSION

We have demonstrated the promoting role of metabolic reprogramming induced by CDK12 deficiency in the progression and treatment resistance of CRPC. Mechanistically, CDK12 deficiency leads to aberrant activation of the mitochondrial ETC in CRPC cells, greatly enhancing cellular energy conversion and utilization efficiency. Additionally, we have shown that CDK12 defect causes a transcriptional instability of ACSL4 in CRPC cells, suppressing its protein expression and blocking the occurrence of ferroptosis by preventing the entry of long‐chain PUFAs into the cell membrane. More translationally, we have identified the mitochondrial ETC complex as a potential new target for treating CDK12‐deficient CRPC, providing new insights into the treatment for this CRPC subtype.

## AUTHOR CONTRIBUTIONS

HH led the study by designing and interpreting results, obtaining funding and reviewing the manuscript. HZ participated in performing the experiments, analysing results, conducting bioinformatics analysis and writing manuscripts. YZ and YC participated in performing the experiments and collecting the data. YF participated in conducting the bioinformatics analysis. WH, ZX, YFZ and QW participated in the manuscript discussion. YY and JG participated in interpreting and reviewing the manuscript's results.

## FUNDING INFORMATION

This study was supported by the National Natural Science Foundation of China (82173131 to HH, 81972414 to YY and 82260500 to JG), Science and Technology Innovation Commission of Shenzhen Municipal Government Grants (JCYJ20210324104007022 to HH), the Natural Science Foundation of Guangdong Province (2023A1515010124 to HH) and the Key Natural Science Research Project of Anhui Universities (2023AH053332 to YY).

## CONFLICT OF INTEREST STATEMENT

All authors declare no conflict of interest related to this manuscript.

## ETHICS STATEMENT

This study was carried out in accordance with the recommendations of Southern University of Science and Technology Laboratory Animal Welfare Ethics Review Committee.

## Supporting information

Supporting Information

Supporting Information

## Data Availability

All data generated or analysed during this study are included in Supplementary Data. The RNA‐seq datasets generated during this study are available in the Gene Expression Omnibus repository (GSE246983).
