# Supplementary material for: Cyclin‐dependent kinase 12 deficiency reprogrammes cellular metabolism to alleviate ferroptosis potential and promote the progression of castration‐resistant prostate cancer
Source: Clin Transl Med. 2024 May 12;14(5):e1678. doi: 10.1002/ctm2.1678 (PMC11089090; doi:10.1002/ctm2.1678)
Supplement: Supplementary file 1 — Supporting Information [file CTM2-14-e1678-s002.dotx]

Supplementary material

CDK12 Deficiency Reprograms Cellular Metabolism to Alleviate Ferroptosis Potential and Promote the Progression of Castration-Resistant Prostate Cancer

Haozhe Zhang^#^, Yi Zhou^#^, Yating Feng, Wenli Hou, Yafei Chen, Zengzhen Xing, Yifan Zhang, Qiang Wei, Yu Yin^*^, Ju Guo^*^, Hailiang Hu^*^


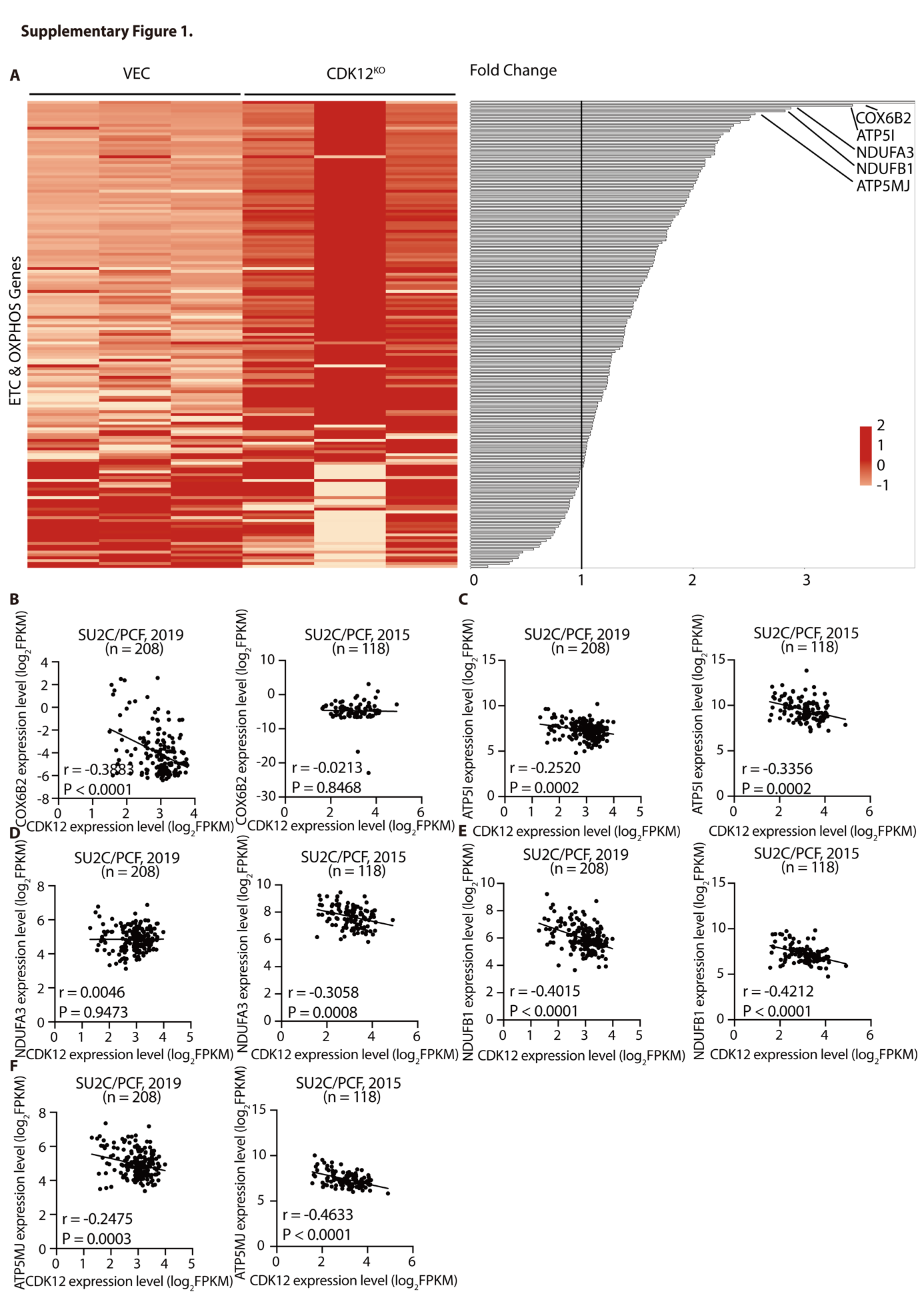


**Figure. S1. CDK12 deficiency reprograms cellular energy metabolism. (A)** In the C4-2 VEC and CDK12^KO^ cell RNA-seq data, heatmap of expression levels of mitochondrial ETC and oxidative phosphorylation-related genes and fold changes in expression (CDK12^KO^ vs. VEC). Data were subjected to a Student t-test. **(B-F)** A positive correlation between mitochondrial ETC-related genes and CDK12 mRNA expression in CRPC patients. Data were analyzed using Pearson correlation analysis.


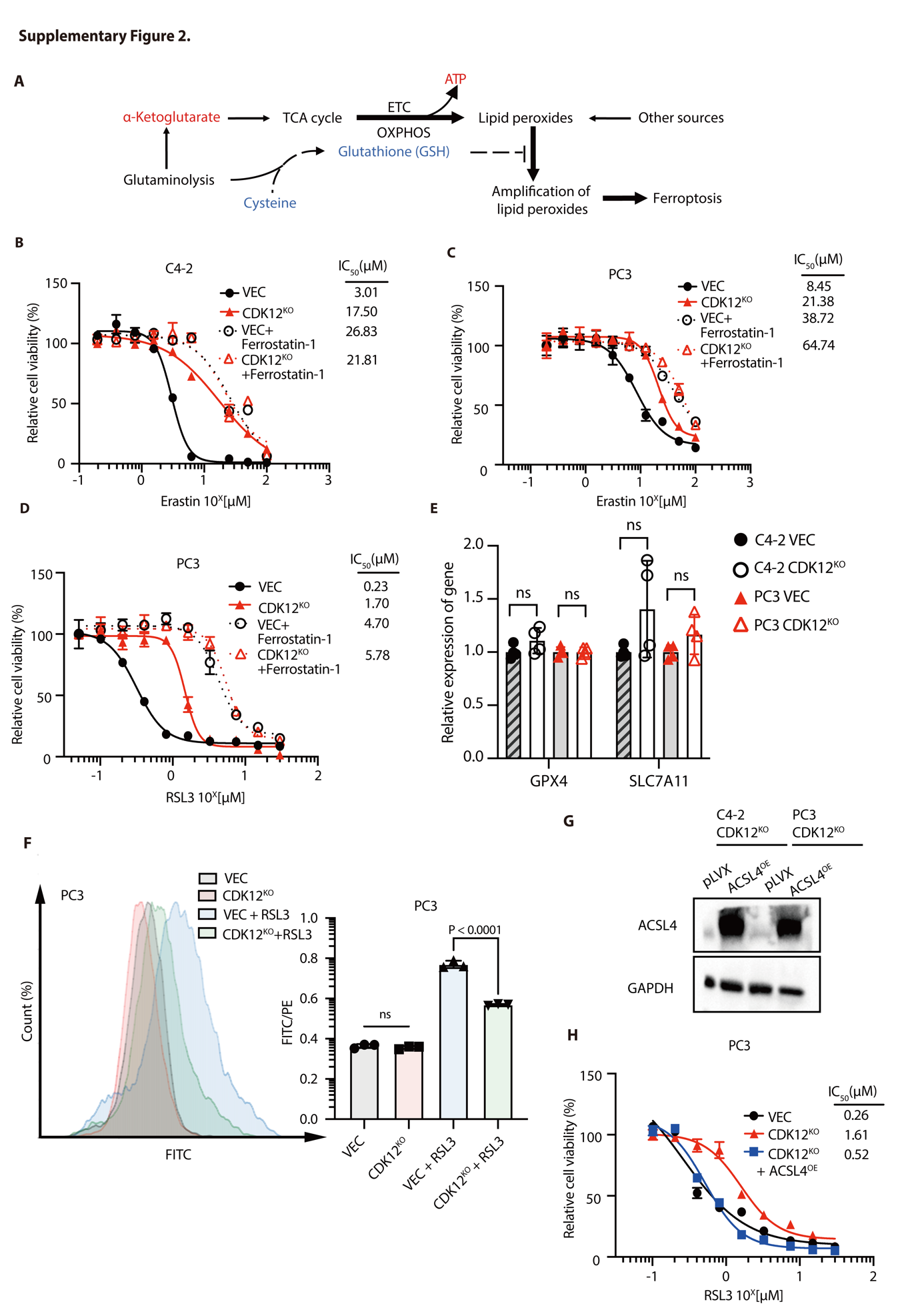


**Figure. S2. CDK12-deficient CRPC cells escape ferroptosis by downregulating ACSL4 expression. (A)** CDK12 deficiency reprograms cellular energy metabolism. Red represents metabolites with elevated levels after CDK12 knockout, and blue represents metabolites with reduced levels after CDK12 knockout. **(B)** Treating C4-2 VEC cells and C4-2 CDK12^KO^ cells with Erastin with or without ferrostatin-1 (2μM) for 48 hours using a concentration gradient (n = 3 biologically independent repeat). The cell count was characterized using the chemiluminescence assay, and the IC50 value was calculated using GraphPad Prism10. **(C-D)** Treating PC3 VEC cells and PC3 CDK12^KO^ cells with RSL3 or Erastin with or without ferrostatin-1 (2μM) for 48 hours using a concentration gradient (n = 3 biologically independent repeat). **(E).** The expression levels of *GPX4* and *SLC7A11* were detected using qRT-PCR (n = 4 parallel repeat). The experiments were repeated in three biologically independent cell experiments. **(F)** Membrane ROS levels were lower in CDK12 knockdown PC3 cells after treatment with RSL3. Treating cells with 2μM RSL3 or fluid (DMSO) for 12 hours. Cell membrane ROS levels were measured using the BODIPY-C11 probe, and the fluorescence signals were analyzed and quantified using flow cytometry (n = 3 biologically independent repeat). **(G-H)** Overexpression of ACSL4 restored the sensitivity of CDK12 knockout CRPC cells to ferroptosis inducers. Treating cells with RSL3 for 48 hours using a concentration gradient (n = 3 biologically independent repeat). Data were shown as means ± s.d. and subjected to an Unpaired t-test, ns means P > 0.05.


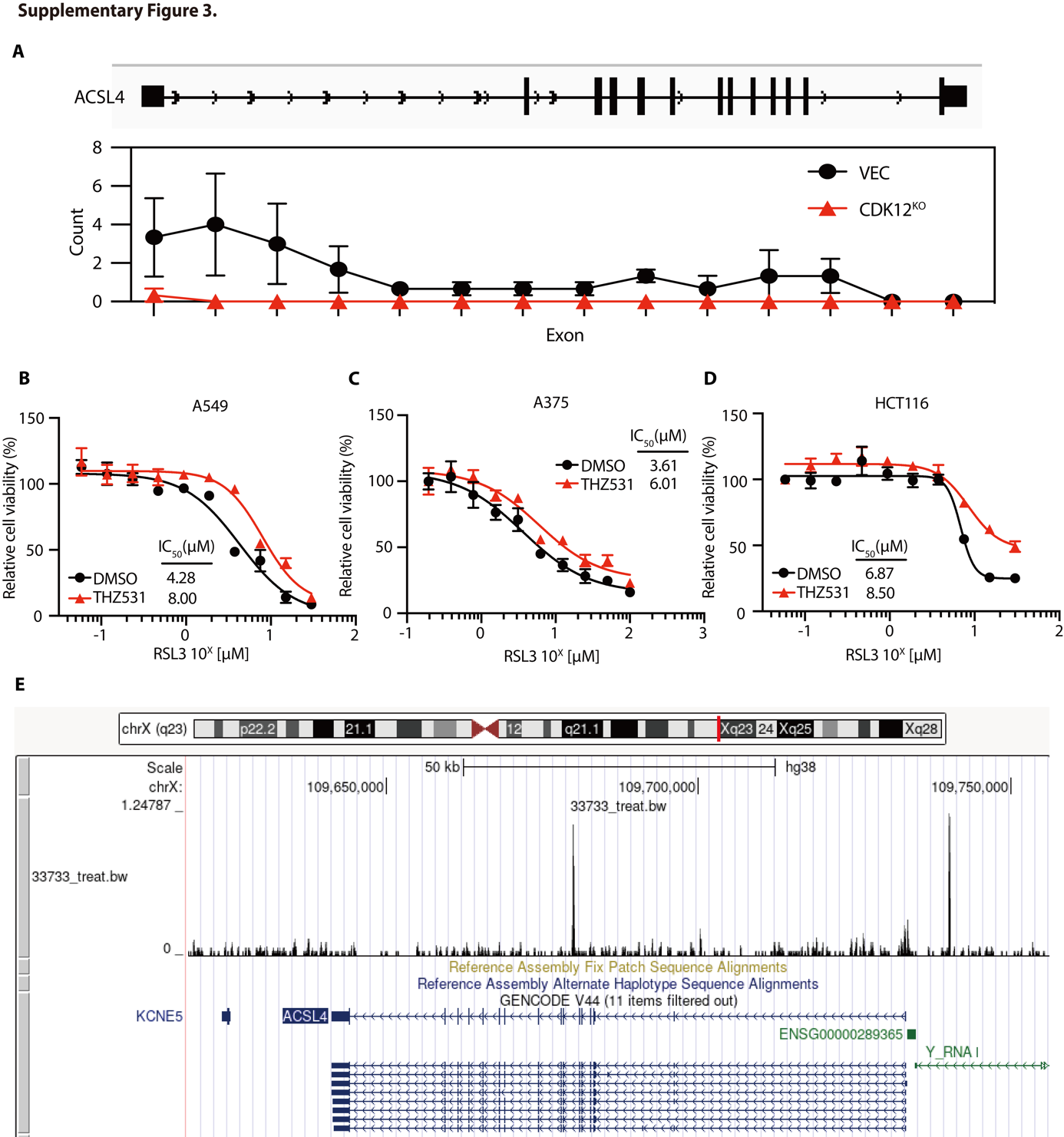


**Figure. S3. The downregulation of ACSL4 expression by CDK12 is dependent on its kinase activity. (A)** In the C4-2 VEC and CDK12 KO cell RNA-seq data, the expression level of ACSL4’s all exons. **(B-D).** Inhibition of CDK12 kinase activity by THZ531 renders different cancer cells resistant to ferroptosis inducer RSL3. Treating cells with RSL3 with or without THZ531 for 48 hours using a concentration gradient (n = 3 biologically independent repeat). **(E).** The chromatin immunoprecipitation results of RNA Pol II in LNCaP cells show binding peaks near the sixth exon of the ACSL4 chromatin region. Data were shown as means ± s.d.


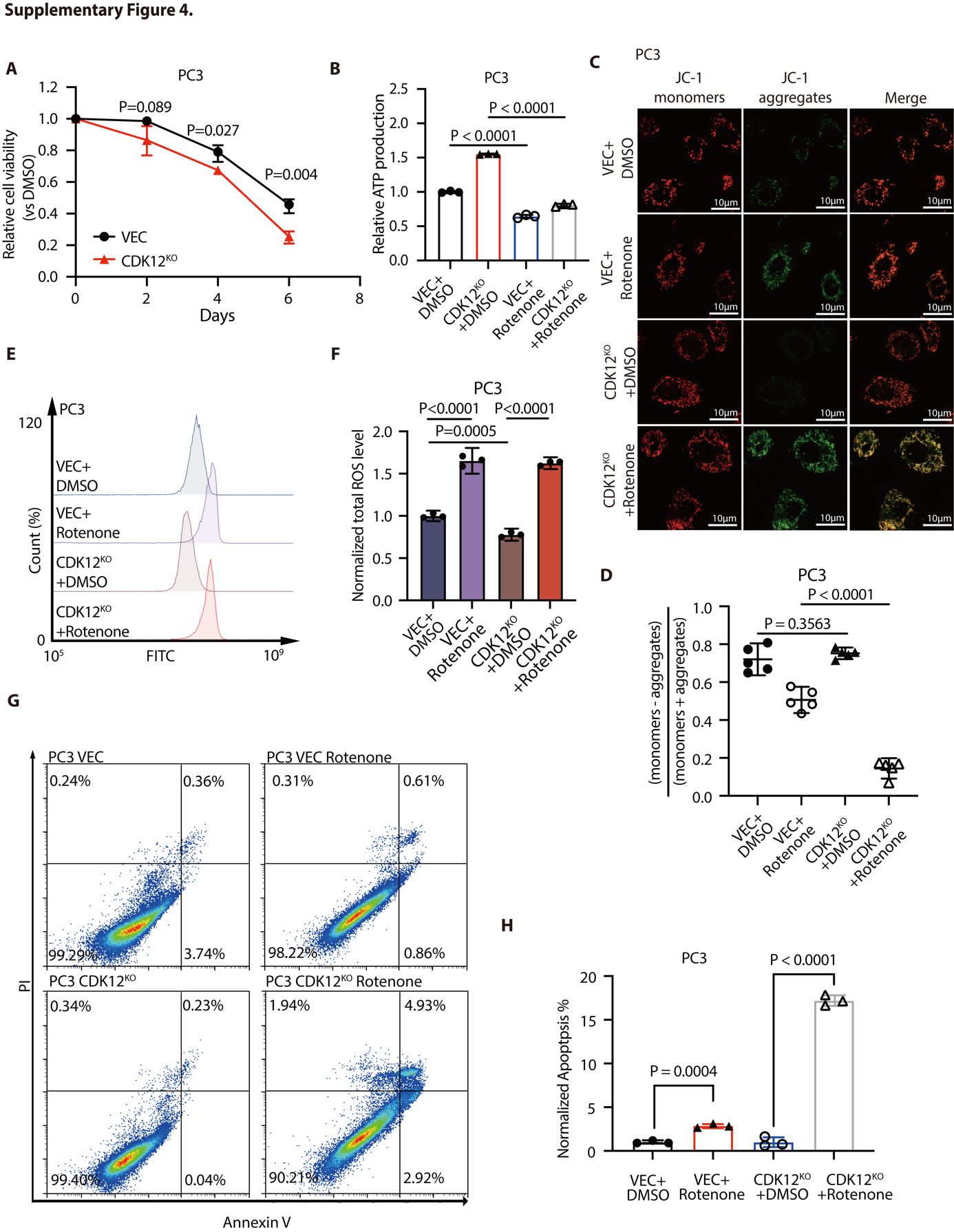


**Figure. S4. CDK12-deficient CRPC exhibits a heightened dependence on the functionality of ETC and is more sensitive to ETC inhibitors. (A)** Rotenone inhibited the growth of PC3 cells effectively (n = 3 biologically independent repeat). **(B)** After knocking out CDK12, the ATP levels in PC3 cells increase. Rotenone can inhibit the production of ATP in CRPC cells, and it is more effective in cells with CDK12 knockout (n = 3 biologically independent repeat). **(C-D)** Rotenone can disrupt the mitochondrial membrane potential of PC3 cells, and this disruption is more pronounced in cells with CDK12 knockout. The mitochondrial membrane potential was characterized using the JC-1 probe and observed using confocal microscopy. Monomers represent average mitochondrial membrane potential, while aggregates represent the loss of mitochondrial membrane potential (n = 3 biologically independent repeat). Quantifying fluorescent signals using image J (imagej.nih.gov). **(E-F)** Rotenone caused elevated total ROS levels in PC3 cells. Total ROS levels were detected using the DCFH-DA probe, and the fluorescence signals were analyzed and quantified using flow cytometry (n = 3 biologically independent repeat). **(G-H)** Rotenone can induce apoptosis in PC3 cells, and cells with CDK12 knockout have a higher percentage of apoptosis. Flow cytometry was used to separate and quantify the cells (n = 3 biologically independent repeat). Data were shown as means ± s.d. and subjected to an Unpaired t-test, ns means P > 0.05.


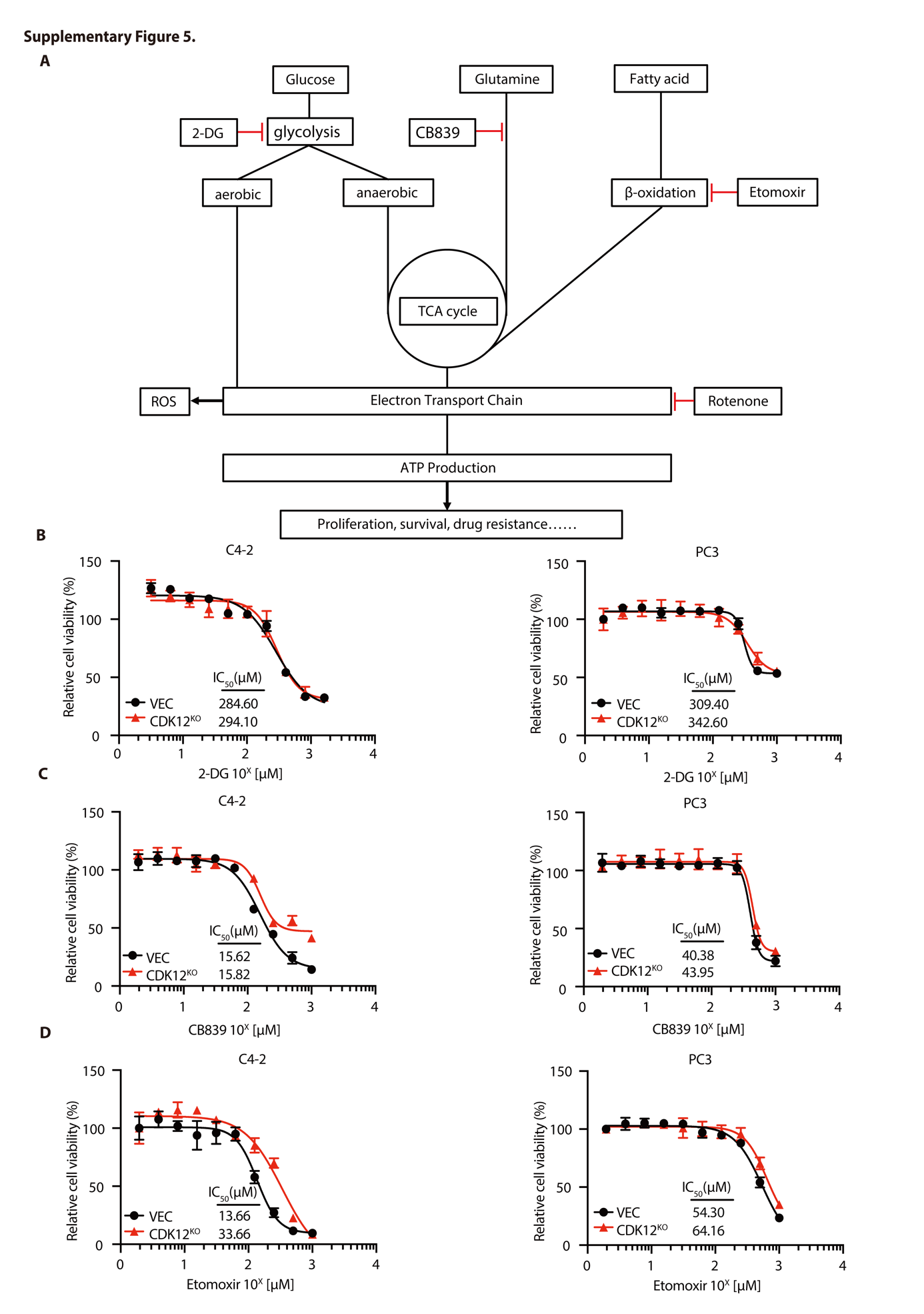


**Figure. S5. CDK12-deficient CRPC exhibits a heightened dependence on the functionality of ETC. (A)** The mitochondrial ETC is the center of cellular energy metabolism, and different drugs affect cellular energy metabolism by inhibiting different pathways. **(B-D)** Treating cells with 2-DG, CB839, and Etomoxir for 48 hours using a concentration gradient (n = 3 biologically independent repeat). Data were shown as means ± s.d.


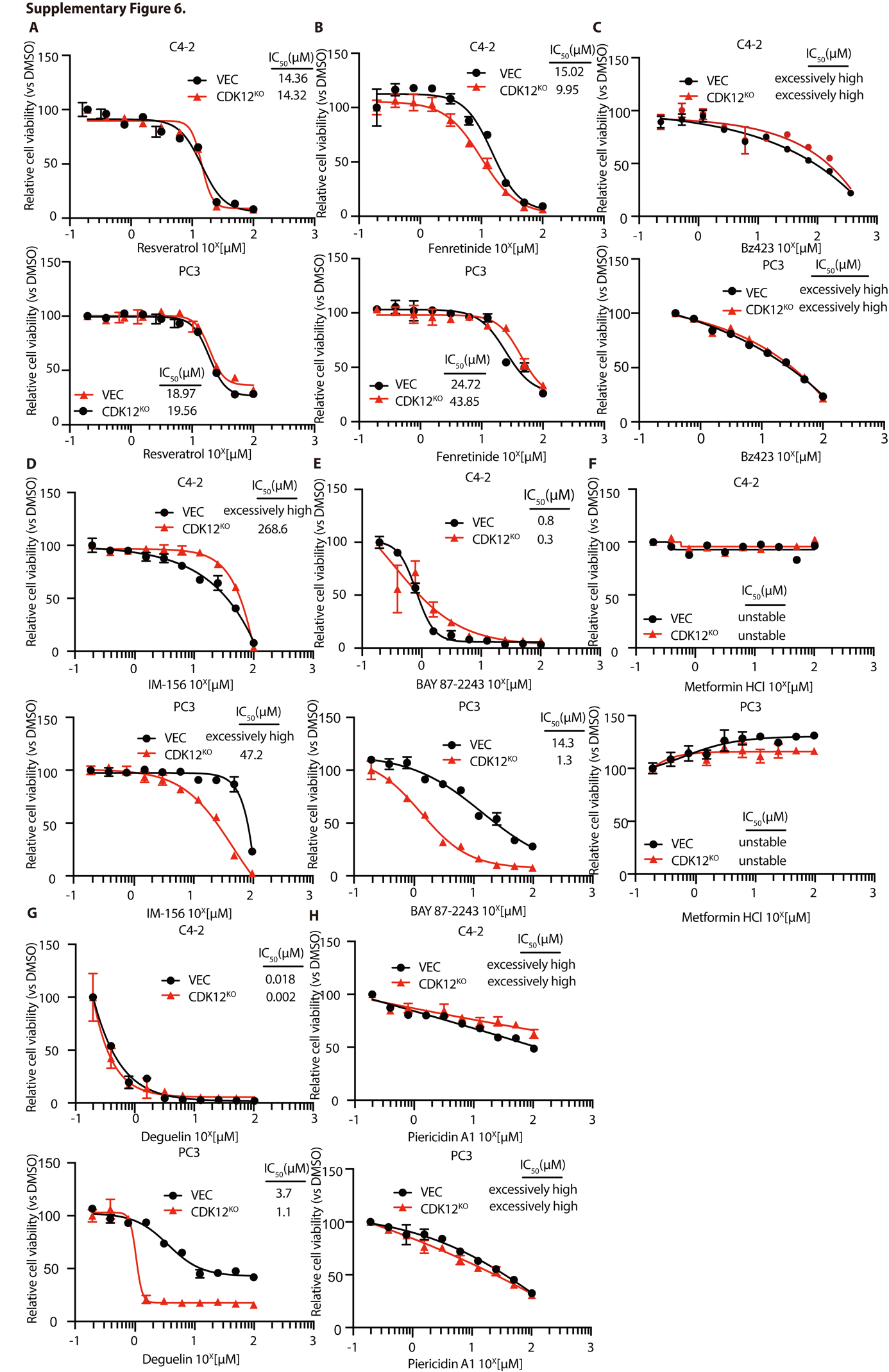


**Figure. S6. Mitochondrial ETC inhibitors suppress mCRPC tumors in vitro. (A-H)** Treating CRPC cells with different metabolic therapeutics drugs for 48 hours using a concentration gradient (n = 3 biologically independent repeat). Data were shown as means ± s.d.

Supplementary Table 1. qRT-PCR primer sequence.

| *ACTB*-F | CATGTACGTTGCTATCCAGGC | *ACTB*-R | CTCCTTAATGTCACGCACGAT |
| --- | --- | --- | --- |
| *ACSL4*-F | CATCCCTGGAGCAGATACTCT | *ACSL4*-R | TCACTTAGGATTTCCCTGGTCC |
| *COX6B2*-F | TTCCTGGACTACCACCGCT | *COX6B2*-R | CGGCGAAAATCCCGTTCTTG |
| *ATP5I*-F | CAGGTCTCTCCGCTCATCAAG | *ATP5I*-R | GCCCGAGGTTTTAGGTAATTGT |
| *NDUFA3*-F | GGGGCCTCGCTGTAATTCTG | *NDUFA3*-R | GACGGGCACTGGGTAGTTG |
| *NDUFB1*-F | GTCCCTATGGGATTTGTCATTGG | *NDUFB1*-R | CAGTTAGCCGTTCATCACTCTT |
| *ATP5MJ*-F | ATTGTATGGAACTCGCGGGT | *ATP5MJ*-R | TCTTTTATCAGCAGCCCGGA |
| *GCLC*-F | GGAGACCAGAGTATGGGAGTT | *GCLC*-R | CCGGCGTTTTCGCATGTTG |
| *GCLM*-F | CATTTACAGCCTTACTGGGAGG | *GCLM*-R | ATGCAGTCAAATCTGGTGGCA |
| *GPX4*-F | GAGGCAAGACCGAAGTAAACTAC | *GPX4*-R | CCGAACTGGTTACACGGGAA |
| *SLC7A11*-F | TCTCCAAAGGAGGTTACCTGC | *SLC7A11*-R | AGACTCCCCTCAGTAAAGTGAC |
